# Polygenic scores for executive functioning as predictors of performance improvements after repeated testing in major psychiatric disorders

**DOI:** 10.1038/s41598-026-41345-1

**Published:** 2026-03-16

**Authors:** Alba Navarro-Flores, Maria Heilbronner, Hajar Rafiee, Bernadette Wendel, Sergi Papiol, Kristina Adorjan, Monika Budde, Mojtaba Oraki Kohshour, Eva C. Schulte, Daniela Reich-Erkelenz, Fanny Senner, Ion-George Anghelescu, Volker Arolt, Bernhard T. Baune, Udo Dannlowski, Detlef E. Dietrich, Andreas J. Fallgatter, Christian Figge, Fabian U. Lang, Georg Juckel, Carsten Konrad, Jens Reimer, Eva Z. Reininghaus, Max Schmauß, Andrea Schmitt, Carsten Spitzer, Jens Wiltfang, Jörg Zimmermann, Peter Falkai, Thomas G. Schulze, Urs Heilbronner

**Affiliations:** 1 Institute of Psychiatric Phenomics and Genomics (IPPG), LMU University Hospital, LMU Munich, Munich, Germany; 2 International Max Planck Research School for Translational Psychiatry (IMPRS-TP), Munich, Germany; 3 School of Psychology, University of Padua, Padua, Italy; 4 Department of Genetic Epidemiology, University Medical Center Göttingen, Georg-August-University, Göttingen, Germany; 5 Max Planck Institute of Psychiatry, Munich, Germany; 6 University Hospital of Psychiatry and Psychotherapy, University of Bern, Bern, Switzerland; 7 Department of Immunology, Faculty of Medicine, Ahvaz Jundishapur University of Medical Sciences, Ahvaz, Iran; 8 Department of Psychiatry and Psychotherapy, Faculty of Medicine and University Hospital Bonn, University of Bonn, Bonn, Germany; 9 Institute of Human Genetics, Faculty of Medicine and University Hospital Bonn, University of Bonn, Bonn, Germany; 10 German Center for Mental Health (DZPG), partner site Munich-Augsburg, Munich, Germany; 11 Centres for Psychiatry Suedwuerttemberg, Ravensburg, Germany; 12 Department of Psychiatry and Psychotherapy, LMU University Hospital, LMU Munich, Munich, Germany; 13 Department of Psychiatry and Psychotherapy, Mental Health Institute Berlin, 14050 Berlin, Germany; 14 Institute for Translational Psychiatry, University of Münster, 48149 Munster, Germany; 15 Department of Psychiatry, University of Münster, 48149 Munster, Germany; 16 Department of Psychiatry, Medical School and University Medical Center OWL, Protestant Hospital of the Bethel Foundation, Bielefeld University, Bielefeld, Germany; 17 German Center for Mental Health (DZPG), Site Jena Magdeburg Halle, Magdeburg, Germany; 18 Center for Intervention and Research on Adaptive and Maladaptive Brain Circuits Underlying Mental Health (C-I-R-C), Site Jena Magdeburg Halle, Magdeburg, Germany; 19 AMEOS Clinical Center Hildesheim, 31135 Hildesheim, Germany; 20 Department of Psychiatry and Psychotherapy, Tübingen Center for Mental Health (TüCMH), University of Tübingen, 72076 Tübingen, Germany; 21 German Center for Mental Health (DZPG), partner site Tübingen, Tübingen, Germany; 22 Karl-Jaspers Clinic, European Medical School Oldenburg-Groningen, 26160 Oldenburg, Germany; 23 Department of Psychiatry II, Ulm University, Bezirkskrankenhaus Günzburg, 89312 Günzburg, Germany; 24 Department of Psychiatry, Ruhr University Bochum, LWL University Hospital, 44791 Bochum, Germany; 25 Department of Psychiatry and Psychotherapy, Agaplesion Diakonieklinikum, 27356 Rotenburg, Germany; 26 Department of Psychiatry and Psychotherapy, University Medical Center Hamburg-Eppendorf, 20246 Hamburg, Germany; 27 Division of Psychiatry and Psychotherapeutic Medicine, Research Unit for Bipolar Affective Disorder, Medical University of Graz, 8036 Graz, Austria; 28 Clinic for Psychiatry, Psychotherapy and Psychosomatics, Medical Faculty, Augsburg University, Bezirkskrankenhaus Augsburg, 86156 Augsburg, Germany; 29 Laboratory of Neuroscience (LIM27), Institute of Psychiatry, University of Sao Paulo, São Paulo, SP 05453-010 Brazil; 30 Department of Psychosomatic Medicine and Psychotherapy, University Medical Center Rostock, 18147 Rostock, Germany; 31 Department of Psychiatry and Psychotherapy, University Medical Center Göttingen, 37075 Göttingen, Germany; 32 Psychiatrieverbund Oldenburger Land gGmbH, Karl-Jaspers-Klinik, 26160 Bad Zwischenahn, Germany; 33 Department of Psychiatry and Behavioral Sciences, SUNY Upstate Medical University, Syracuse, NY USA; 34 Department of Psychiatry and Behavioral Sciences, Johns Hopkins University School of Medicine, Baltimore, MD USA; 35 Department of Psychiatry, Melbourne Medical School, The University of Melbourne, Melbourne, Australia; 36 The Florey Institute of Neuroscience and Mental Health, The University of Melbourne, Parkville, VIC, Australia; 37 Center for Systems Neuroscience (ZSN), 30559 Hannover, Germany; 38 Department of Psychiatry, Medical School of Hannover, 30625 Hannover, Germany; 39 Center for Psychosocial Medicine, Academic Teaching Hospital Itzehoe, Itzehoe, Germany; 40 German Center for Neurodegenerative Diseases (DZNE), 37075 Göttingen, Germany; 41 Neurosciences and Signaling Group, Institute of Biomedicine (iBiMED), Department of Medical Sciences, University of Aveiro, Aveiro, Portugal

**Keywords:** Genetics, Executive function, Performance improvement, Major psychiatric disorders, Polygenic scores, Practice effects, Diseases, Genetics, Medical research, Neurology, Neuroscience, Psychology, Psychology, Risk factors

## Abstract

Executive Functions (EF) control goal-oriented behaviors and are disrupted in patients with psychiatric disorders (PPD). Repeated EF testing in clinical practice and research aims to evaluate disease progression and treatment efficacy. Performance improvements (or “practice effects”) are expected, while deficiencies had been proposed as markers of conversion to dementia. Given the high conversion risk in PPD, understanding the determinants of practice effects is of high relevance. However, the role of genetic predisposition remains unknown. We aim to test the influence of polygenic scores on practice effects across PPD. Data were drawn from the longitudinal deep-phenotyping PsyCourse Study (*N* = 1,558). Latent factor analysis revealed a common Executive Function factor (cEF), which explained shared variability of 5 different EF tests. We tested the association of polygenic scores for the cEF (PGS-cEF) – based on the largest GWAS on cEF to date (*N* = 427,037) - with cEF scores improvements in PsyCourse. The PGS for general psychopathology “p-Factor” (PGS-PF) were used as a psychiatric liability comparison. Volunteer adults (> 18 y.o.), with and without diagnoses within the Affective-Psychotic spectrum were recruited across Germany and Austria, followed-up four times at six months intervals. The mean PGS-cEF was lower in patients with psychotic vs. affective disorders (diff: -0.01, 95% CI -0.02 to -0.002) and controls (diff: -0.02, 95% CI -0.03 - -0.006); while the mean PGS-PF was higher in psychotic vs. affective disorders (diff: 0.02, 95% CI 0.01 to 0.04) and controls (diff: 0.03, 95% CI 0.02–0.05). Overall, the PGS-cEF x Visit interaction was associated with the phenotypic latent cEF scores (eta^2^ = 0.03; *p* = 1.32e-15), but not with the scores of individual EF tests. There was no PGS-PF x Visit interaction associated with any cognitive score. To the best of our knowledge, this is the first analysis of genetic factors related to EF improvements in PPD. The PGS-cEF, but not the PGS-PF predicted practice effects in cEF scores over 18 months. This prediction was only observable using the latent‑cEF level, and not with the individual scores. Genetic testing and deep phenotyping might hint further information about EF vulnerability in PPD.

**Supplementary Information:**

The online version contains supplementary material available at 10.1038/s41598-026-41345-1.

## Introduction

Executive functions (EF) are high-level cognitive processes that guide goal-directed behaviors. Core domains of EF comprise cognitive flexibility, inhibition control and working memory^2^. These abilities are usually assessed using EF tests, and the shared variability among them can be explained by a top-level latent factor, known as common executive function factor (cEF)^3^. This characteristic of EF is consistent with the well-validated “Unity and Diversity” model, which postulates that EF share common features (unity) while remaining distinct in their specific abilities (diversity)^3^.

EF operate in an integrated hierarchical system with the top-level cEF correlated with specific separable components that interdependently control other cognitive domains^3^. However, this structure of EF is often overlooked when they are evaluated using the results of single EFs tests, because each test simultaneously assesses a variety of related abilities, such as visuo-spatial or verbal skills. This has been dubbed the “task impurity” challenge^3^. For each EF test, this impurity appears unavoidable, since each lower-level task that EF control is simultaneously assessed. This emphasizes the importance of the latent level “unity” cEF as a phenotype that aggregates EF components only partially observed using individual EF tests.

While EF deficits have been originally described in individuals with acquired brain damage^4^, they are also prevalent in psychiatric disorders^5^. EF deficits are not only a consequence of psychiatric symptoms but also could act as a predictor of their progression over time^6^. As EF deficits worsen, psychiatric symptoms tend to worsen as well, highlighting the importance of addressing EF deficits in the treatment of psychiatric disorders. Furthermore, there is evidence regarding the effectiveness of cognitive remediation interventions in adults^7^, hinting that the plasticity of executive functioning represents a potential target for rehabilitation.

Repeated testing carries on practice effects that, in healthy participants, could theoretically be explained by the potential actual enhancement of the (latent) constructs, the reduction of construct irrelevant confounders (e.g., anxiety), or the development of solving strategies development^8,9^. Empirical studies have provided evidence for these hypotheses, supporting mostly the latter two explanations^6,7^. However, the exact nature of the factors involved in improvements of EF scores after repeated testing is not clear, and these effects are commonly found in neurotypical individuals^10^.

Deficiency in profiting from these practice effects hints to cognitive impairment especially in patients with neurological disorders^11^. A systematic review of 27 studies showed that smaller and less-robust practice effects correlated with current or future cognitive impairment or dementia diagnosis, hence have been proposed as a marker of dementia risk^12^. Given that patients with major psychiatric disorders are already at-risk population for this outcome^13^, understanding the determinants of these markers is of high relevance for the field. In schizophrenia, the magnitude of practice effects significantly reduced compared to neurotypical individuals^14^. Notably, older adults with schizophrenia failed to benefit from repeated testing, as evidenced by a lack of improvement in their cognitive performance compared to their age-comparable controls^15^. A similar finding was observed in a separate study, which demonstrated that poorer cognitive performance at baseline and a longer duration of illness were associated with reduced retesting effects in patients with schizophrenia^16^. In patients with Alzheimer’s Disease and related dementias, reduced practice effects were linked to a more rapid progression of cognitive decline over a two-year period^17^. These findings underscore the importance of investigating performance improvements in EF after repeated testing, particularly in the context of psychiatric research and clinical practice.

Regarding the heritability of EF, behavioral genetic studies have consistently shown that the latent cEF is highly heritable, whereas individual EF tests exhibit only moderate heritability^18^. This could be explained because latent factors aggregate measurement information across multiple tasks, reducing measurement error and increasing reliability. This pattern is expected when measurement error inflates phenotypic variance in individual tests, thereby suppressing heritability estimates^19^. Importantly, while EF and general intelligence (g), significant degree of overlap, they are distinct constructs at the phenotypic, genetic, and molecular levels^20,21^. Despite being genetically correlated (r_g_ = 0.766)^22^, specific single nucleotide polymorphisms (SNPs) associated with the cEF were found to be enriched in genes related to synaptic processes, such as the *GABA*_*A*_ or voltage-gated potassium channel. These findings contrast with the results of the GWAS of general intelligence, which identified genes enriched in medium spiny and pyramidal neurons^23^. These results highlight the distinct neural basis of EF and general intelligence, emphasizing the importance of considering these constructs as separate entities in research and clinical practice.

The longitudinal course of EF has been linked to alterations in prefrontal cortex networks^24^, aging, and the presence of psychiatric disorders^25^. However, the short-term dynamics of EF remain poorly understood. A recent longitudinal GWAS conducted by our group investigated the relationship between practice effects and genetic variation in a single EF test (the Trail-Making-Test Part B)^26^. The results suggested that polymorphisms at rs150547358, located in the intron region of ring finger protein 180 (RNF180), involved in serotonin metabolism, may influence practice effects. However, it remains unclear whether a polygenic score for EF that aggregates multiple genetic variants can predict short-term changes in EF performance.

To address this question, we examined the effects of polygenic scores for a latent cEF on the improvement of scores on various EF tests following repeated assessments. We compared these effects to those of polygenic scores for general psychopathology (“P factor”), which are negatively correlated with EF performance^27^. Our results show that differences in genetic predisposition for EF are associated with EF performance improvement in adults with and without major psychiatric disorders. Notably, these improvements are more pronounced when a latent phenotypic cEF, derived from multiple EF tests, is used as the dependent variable, but not when individual EF tests are used.

## Methods

### Study participants and genotyping

**Fig. 1:**
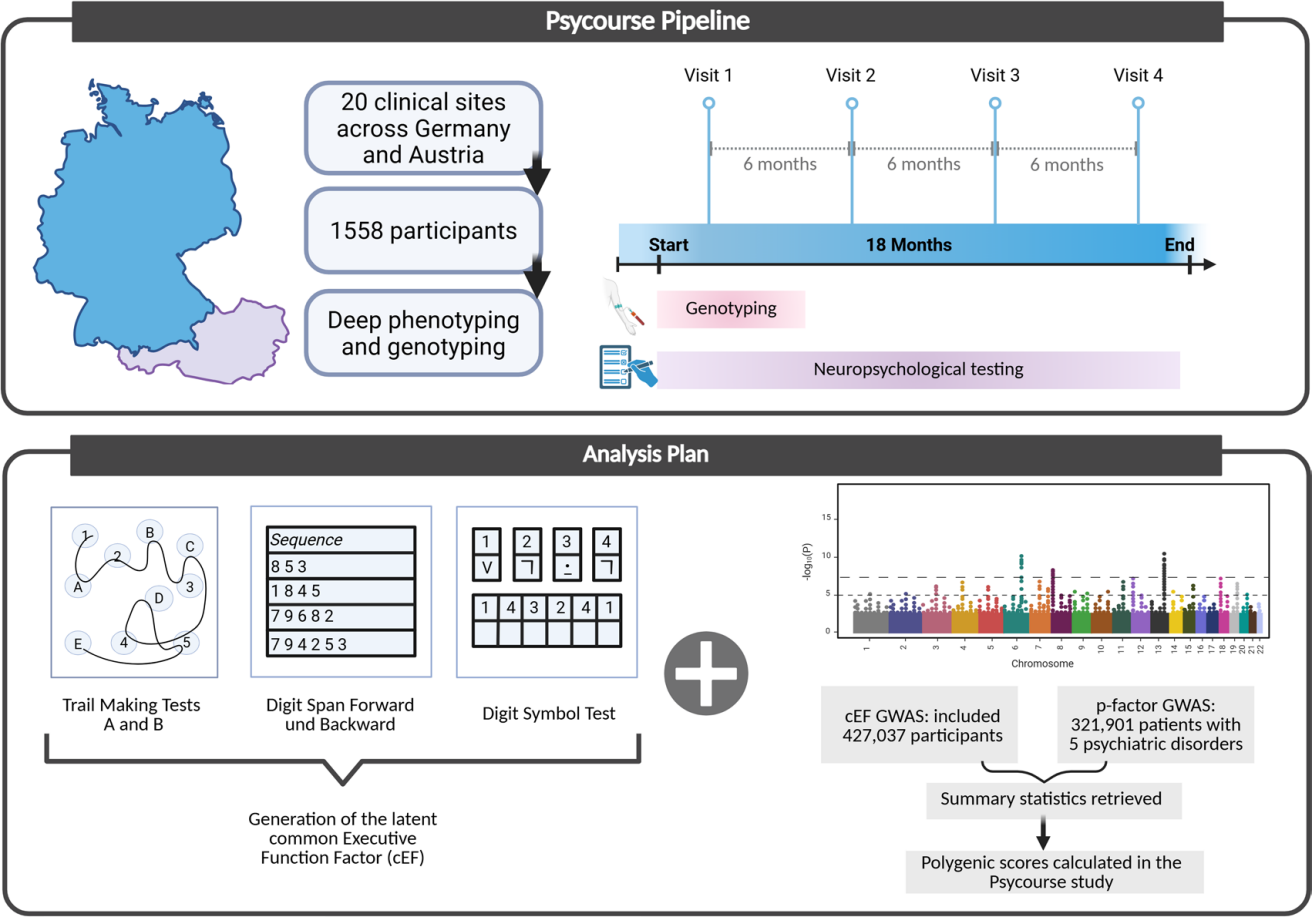
Description of the study. The PsyCourse Study recruited participants with psychiatric disorders (Major Depressive Disorder, Bipolar Disorder, and Schizophrenia) and healthy controls across 20 sites in Germany and Austria. The patients were genotyped and deeply phenotyped, including 5 executive function tests. The current analysis these tests to calculate a latent common Executive Function Factor and used the summary statistics of previous GWA studies on the cEF and the “p-factor” to calculate polygenic scores (PGS) in our cohort. The influence of the PGS on performance improvements over time were analyzed. Created in https://BioRender.com.

The PsyCourse Study is a transdiagnostic deep phenotyping observational study of the psychotic-to-affective spectrum^28^. Our study adheres to the Strengthening the Reporting of Observational studies in Epidemiology – Molecular Epidemiology (STROBE-ME) guidelines, as outlined in eTable 1^29^. The sample was recruited at 20 clinical centers in Germany and Austria, from January 1, 2012, to December 31, 2019. Clinical participants received only treatment-as-usual intervention and were not undergoing cognitive-oriented therapies, such as cognitive remediation. Non-clinical (“healthy”) controls were recruited from the community, in the cities of Göttingen and Munich. They were recruited either by post using address lists obtained from the Residents’ Registration Office, or by advertisements in public areas^28^. Participants underwent assessments up to four times, spaced 6 months apart, tolerating a window of ± 4 weeks. Evaluations conducted outside of this window were excluded from this study. A summary of the methodology used is presented in Fig. 1.

We categorized participants into three broad diagnostic groups: neurotypical controls (no psychiatric diagnosis), affective disorders (recurrent major depressive disorder, bipolar I and bipolar II disorder), and psychotic disorders (schizophrenia, schizoaffective disorders, among others). A detailed description of the diagnostic subgroups and their sample sizes is included in eTable 2.

Genotyping was performed using SNP arrays, and the quality control (QC) process is detailed in eMethods 1 (Supplement). Additional information on genotyping can be found in previous publications^30,31^. Due to the sample being exclusively of German-Austrian ancestry, the first two genetic principal components (PC) explained most of the variability in that regard. Hence we selected the first four PC as relevant for statistical analysis.

### Ethical procedures

The PsyCourse study protocol followed the Helsinki Declaration and was approved by the institutional review boards (IRBs) at each clinical site, accordingly^28^. To ensure participant confidentiality, all data were pseudonymized, and a unique identifier was assigned to each participant. This identifier was used to link de-identified data across assessments, allowing for longitudinal analysis of participant outcomes^28^.

### Neuropsychological tests

Neuropsychological assessments were administered by trained raters (primarily research psychologists and psychiatrists) during each of the four visits, using paper-based formats. The individual executive function (EF) tests employed were: Trail Making Test A (TMT-A), which assesses psychomotor speed in the context of a complex cognitive task. Trail Making Test B (TMT-B), which evaluates executive function in the context of a complex cognitive task. Digit Span Forwards (DSP-FW), which measures short-term memory in the context of a working memory task. Digit Span Backwards (DSP-BCK), which assesses working memory in the context of a complex cognitive task. Digit-Symbol Test (DST), which evaluates processing speed in the context of a visual-motor task. To facilitate comparison across tests, the scores for TMT-A and TMT-B were reversed using a logarithmic transformation, such that higher scores indicated better performance. A detailed description of the individual tests and the domains included is presented in eMethods 2.

These tests were selected due to their shared variability which has been suggested in previous studies to construct the latent common EF factor^3,22^. Specifically, they assess the three most common domains included in latent factor analysis of EF unity: cognitive flexibility, inhibition control, and working memory^32^. The correlation between these tests across the four timepoints was confirmed (eFigure 1). Notably, the EF scores improved over time (eTable 3), a finding consistent with our previous report in the control sample^10^.

### Calculation of the latent phenotypic cEF score

The five neuropsychological tests described above were used to compute the latent cEF scores. Although not all of the tests measure exclusively EF, each captures different EF components and therefore contributes significantly to the construction of the latent score. Prior studies that extracted this latent factor have employed comparable neuropsychological batteries^22^.

**Fig. 2:**
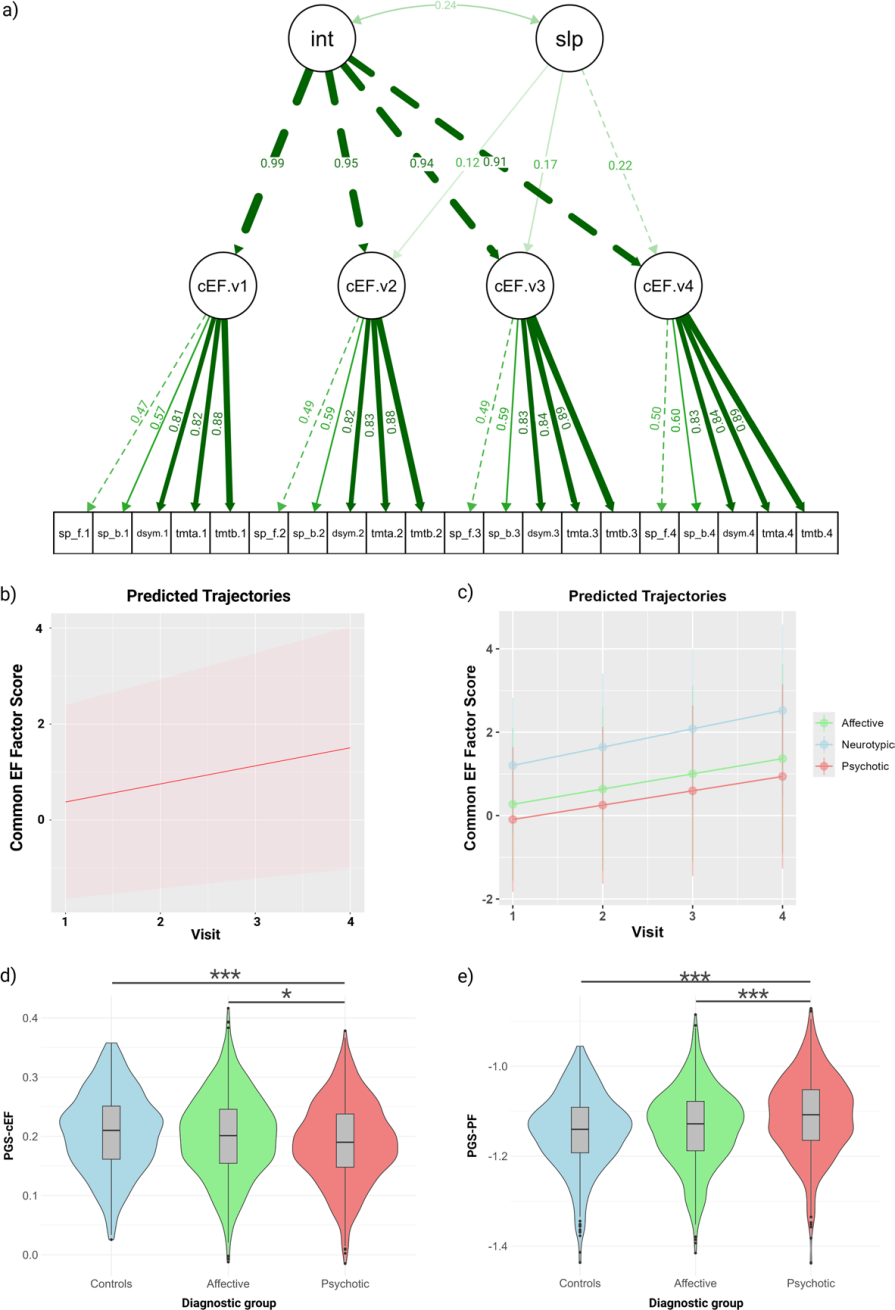
Characteristics of the latent cEF scores and the polygenic scores. (a) Longitudinal confirmatory factor analysis (CFA) measurement model for the common Executive Function (cEF) factor across four visits (v1–v4). Observed indicators represent the five EF tests measured at each assessment: sp_f (Digit Span Forward), sp_b (Digit Span Backward), dysm (Digit-symbol test), tmta (Trail Making Test A), tmtb (Trail Making Test B). The pattern of the lines connecting the same tests across visits (sp_f.v1 ↔ sp_f.v2, tmta.v1 ↔ tmta.v2, etc.) represent correlated residuals capturing method variance within each test (test format familiarity, administration effects) with their respective loadings. “int” represents the latent intercept factor, related to each person’s baseline level of cEF; its thick arrows to cEF.v1–v4 reflect that the intercept loads strongly and similarly on all four time points (values between 0.91–0.99). “slp” refers to the latent slope factor, representing systematic change in cEF over time; its lighter arrows to cEF.v1–v4 (with increasing load values from left to right) encode the time structure of the trajectory, so that higher scores on “slp” correspond to steeper increases in cEF compared to baseline. The curved double arrow between “int” and “slp” (labeled 0.24) is their covariance, indicating that individuals with higher baseline cEF tend to have slighter higher gains; (b) Overall predicted trajectories, with the mean in the dotted line; (c) Predicted trajectories according to diagnostic group; (d) Distribution of the PGS-cEF according to diagnosis; (e) Distribution of the PGS-PF according to diagnosis.

We performed a longitudinal Confirmatory Factor Analysis (CFA) with the lavaan package in R (Fig. 2a)^33^, testing longitudinal measurement invariance by comparing configural, weak (metric), strong (scalar), and strict models. Although model comparison statistics indicated significant differences, the AIC, the BIC and the CFI values across the four models were highly similar (eTable 4). In the configural invariance model we confirmed that the pattern of factor loadings matrix is equivalent across measurement points. The weak-invariance model established equivalence of the factor’s loadings, the strong-invariance model established equality of the latent means, and the strict-invariance model established equality of the residual variances. Because the strict-invariance model fits the data well, we proceeded with a second-order latent growth model.

Missing data were handled with lavaan’s default full‑information maximum‑likelihood (FIML) estimator. Latent factor scores for the four waves were estimated with the lavPredict function for the four time points whenever a participant had data for at least one test in one of the visits. The model of the latent cEF is presented in Fig. 2a. The predicted trajectories indicated a steady increase in cEF across the overall sample and within each diagnostic group (Fig. 2b, c).

### Calculation of the polygenic scores

The polygenic scores (PGS) were calculated using the PRS-CS method (phi = auto setting)^34^. This method uses Bayesian statistics to apply an inversely correlated continuous shrinkage prior to the effect sizes (ES) of the SNPs, meaning that those with smaller ES (probably due to noise) will receive a higher shrinkage^35^. The PGS for the cEF were derived from a large non-overlapping GWAS study including individuals from the UK Biobank (higher PGS for cEF, better performance)^22^, while PGS for the psychopathology factor (p-factor or PF) were chosen as a control due to the transdiagnostic nature of the sample, and were calculated as previously reported (higher PGS for PF, higher psychopathology)^36,37^. Further details of the calculation of the PGS are described in eMethods 3.

### Statistical analysis

The effects of the PGS-cEF and the PGS-PF were analyzed separately due to the genetic association of the p-Factor and executive functions^27^. Accordingly, we fitted linear mixed-effects models with the lme4 function (maximum-likelihood estimation) for each PGS.

The dependent variable for the main analysis was the predicted latent-cEF score, and for the secondary analysis each of the individual test scores. Fixed effects included chronological age, biologically inferred sex, the first four ancestry principal components, and the interaction between the polygenic scores either for the common executive function factor (PGS-cEF) or the p-Factor (PGS-PF) and the repeated-measure variable visit (study time point). A full description of the equations used is presented in eMethods 4.

Random effects were specified as a participant‑level intercept nested within center to capture the hierarchical sampling design, and a diagnostic‑group intercept to model variation attributable to the three diagnostic categories (controls, affective disorders, psychotic disorders). Biological sex and ancestry were derived from the genotype data; participants whose genetically inferred sex conflicted with self‑reported gender were excluded during quality control. All p‑values were adjusted for multiple testing using the false‑discovery‑rate (FDR) procedure. The main effect of visits reported in eTable 3 was obtained from the same models after removing the polygenic‑score terms. All analyses were conducted in R 4.3.2 using RStudio version 2023.9.1.494^38,39^.

**Conference presentation**. Presented in part at the 54th Behavior Genetics Association Annual Meeting, London, UK, June 26–29, 2024, and at the World Congress of Psychiatric Genetics (WCPG), October 15–19, 2024, in Singapore.

## Results

Genetic data of 1,594 participants was available, of whom 1,558 (97.7%) completed at least one neuropsychological test score during one of the four visits and were therefore included in the analysis. A post-hoc analysis of the participants who had only one assessment compared to those who completed two or more, suggested that the attrition was due to a “missing-at-random” effect and not factors related to cognition. A further description of the results can be found on Supplementary Results. The number of participants with available test scores varied across visits (Table 1). At baseline, the mean cEF score was 0.376 (SD 1.01), while 1.20 (0.812) for the controls, 0.272 (0.916) for the affective, and − 0.09 (0.867) for psychotic disorder groups. The baseline characteristics of the sample are presented in Table 2, and follow-up descriptions can be found in eTables 5, 6 and 7 (Supplement).

**Table 1:** Percentage of available data per test and visit according to diagnosis.

Overall (*n* = 1594)	Visit 1		Visit 2		Visit 3		Visit 4	
	*N*	%	*N*	%	*N*	%	*N*	%
TMT-A	1519	95.3	951	59.7	835	52.4	736	46.2
TMT-B	1466	92	905	56.8	818	51.3	706	44.3
DSP-FW	1481	93	941	59	825	51.8	727	45.6
DSP-BCK	1478	92.7	938	58.8	823	51.6	726	45.6
DST	1459	91.5	930	58.3	814	51.1	723	45.4
Controls (*n* = 404)								
TMT-A	393	97.3	251	62.1	245	60.6	219	54.2
TMT-B	394	97.5	247	61.1	244	60.4	214	53
DSP-FW	392	97	251	62.1	246	60.9	219	54.2
DSP-BCK	392	97	250	61.9	245	60.6	219	54.2
DST	380	94	250	61.9	246	60.9	217	53.7
Affective (*n* = 618)								
TMT-A	587	94.9	367	59.4	309	50	246	39.8
TMT-B	571	92.4	355	57.4	301	48.7	240	38.8
DSP-FW	567	91.8	365	59.1	304	49.2	247	40
DSP-BCK	565	91.4	363	58.7	303	49	246	39.8
DST	564	91.3	356	57.6	302	48.9	243	39.3
Psychotic (*n* = 572)								
TMT-A	539	94.2	333	58.2	281	49.1	271	47.4
TMT-B	501	87.6	303	53	273	47.7	252	44.1
DSP-FW	522	91.3	325	56.8	275	48.1	261	45.6
DSP-BCK	521	91.1	325	56.8	275	48.1	261	45.6
DST	515	90	324	56.6	266	46.5	263	46

TMT-A: Trail Making Test, part A; TMT-B: Trail Making Test, part B; DSP-FW: Digit Span Forward; DSP-BCK: Digit Span Backwards; DST: Digit Symbol Test.

**Table 2:** Baseline characteristics of the sample.

	Total (*N* = 1558)		Controls (*N* = 394)		Affective (*N* = 603)		Psychotic (*N* = 561)	
Females (*n*, %)	755	48.5%	231	58.6%	309	51.2%	215	38.3%
Age								
Mean (SD)	41.3	13.9	36.8	15.2	45.1	13.5	40.4	12.2
Median [Min - Max]	41	18–86	31	18–77	47.0	18–86	41.0	18–73
Educational attainment^a^								
aMean (SD)	4.92	1.62	5.70	1.41	5.07	1.53	4.20	1.55
aMedian [Min - Max]	5	1–7	6	2–7	5	1–7	4	1–7
GAF								
aMean (SD)	62.6	17.4	88.5	7.31	60.6	13.1	53.4	13.0
aMedian [Min - Max]	60.0	4–100	90	62–100	60	20–97	53	4–90
No. of antidepressants								
aMean (SD)	0.4	0.6	0.008	0.09	0.65	0.71	0.31	0.53
aMedian [Min - Max]	0	0–3	0	0–1	1	0–3	0	0–2
No. of antipsychotics								
aMean (SD)	0.97	1.01	0	0	0.89	0.86	1.74	0.89
aMedian [Min - Max]	1	0–5	0	0–0	1	0–5	2	0–5
No. of mood stabilizers								
aMean (SD)	0.3	0.5	0.01	0.10	0.66	0.62	0.13	0.37
aMedian [Min - Max]	0	0–3	0	0–1	1	0–3	0	0–2
No. of other psychiatric medication								
aMean (SD)	0.01	0.11	0	0	0.02	0.16	0.007	0.08
aMedian [Min - Max]	0	0–2	0	0–0	0	0–2	0	0–1
No. of tranquilizers								
aMean (SD)	0.16	0.41	0.01	0.11	0.17	0.42	0.28	0.5
aMedian [Min - Max]	0	0–2	0	0–1	0	0–2	0	0–2
cEF score								
aMean (SD)	0.38	1.01	1.20	0.81	0.27	0.92	-0.09	0.87
aMedian [Min - Max]	0.39	-2.87 to 3.18	1.27	-1.35 to 3.18	0.34	-2.87 to 2.59	-0.06	-2.62 to 2.15
aTMT-A score (Mean, SD)	34.6	16.6	26.2	10.3	35.1	15.9	40.2	18.4
TMT-B score (Mean, SD)	80.1	40.8	58.6	24.5	83.5	42.3	93.1	42.8
DSB (Mean, SD)	6.4	2.3	7.7	2.4	6.18	2.1	5.53	2.03
DSF (Mean, SD)	9.5	2.2	10.4	2.1	9.40	2.1	8.84	2.2
DST (Mean, SD)	-0.1	0.9	0.7	0.8	-0.218	0.868	-0.642	0.727
PGS-cEF (Mean, SD)	0.199	0.069	0.208	0.067	0.201	0.070	0.190	0.068
PGS-PF (Mean, SD)	-1.13	0.088	-1.14	0.084	-1.14	0.086	-1.11	0.089

cEF: latent common Executive Function factor; GAF: Global assessment of Functioning; PGS-cEF: Polygenic Scores for the cEF; PGS-PF: Polygenic scores for the P factor. TMT-A: Trail Making Test, part A; TMT-B: Trail Making Test, part B; DSB: Digit span backwards; DSF: Digit span forwards; DST: Digit Symbol test. ^a^ Educational attainment was calculated as a composite score of basic and higher education years.

### Polygenic scores according to diagnostic group

We conducted an analysis of variance (ANOVA) to examine the differences in mean PGS-cEF scores across diagnostic groups. The results revealed significant differences according to diagnostic group (η^2^ = 0.009; *F*(2, 1557) = 7.54; *p-value* = 0.0004). To determine which group difference drove this effect, we performed a Tukey post-hoc test. The results showed that patients with psychotic disorders had a significantly lower mean PGS-cEF score compared to those with affective disorders (*Mdiff* = -0.01, 95% CI [-0.02, -0.002], *p-value* = 0.017) and neurotypical controls (*Mdiff* = -0.02, 95% CI [-0.03, -0.006], *p-value* = 0.0004).

As expected, the PGS-PF was significantly different according to diagnostic group (η^2^ = 0.03; *F*(2, 1557) = 22.11; *p-value* = 3.39e-10). Specifically, patients with psychotic disorders had a higher mean PGS-PF score compared to participants with affective disorders (*Mdiff* = 0.02, 95% CI [0.01, 0.04], *p-value* = 9e-07) and neurotypical controls (*Mdiff* = 0.03, 95% CI [0.02, 0.05], *p-value* < 1e-07). Notably, no significant differences were found between controls and participants with affective disorders in the means of PGS-cEF and PGS-PF (Fig. 2d-e).

### The impact of PGS-cEF on phenotypic cEF scores over time

**Fig. 3:**
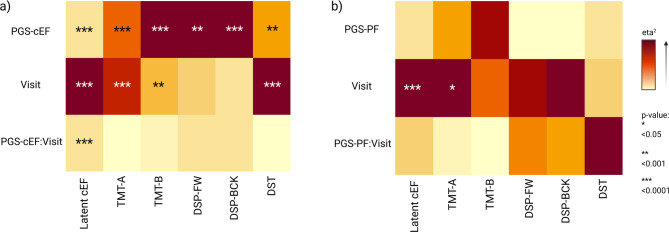
Results of the regression models for the PGS-cEF and PGS-PF. Results of the regression models for the latent cEF scores, and the individual test scores after adjusting for (a) the models adjusting for the PGS-cEF, and (b) the models adjusting for the PGS-PF, where darker color tones indicate larger effect sizes and the stars correspond to statistical significance.

Phenotypic latent cEF scores were significantly associated with the PGS-cEF (η^2^ = 0.3, *p-value* = 1.35e-10), visit (η^2^ = 0.39, *p-value* < 1.32e-15), and the interaction between PGS-cEF and visit (η^2^ = 0.3, *p-value* < 1.32e-15). The PGS-cEF was significantly associated with all the individual test scores (TMT-A η^2^ = 0.008, *p-value* = 7.57e-07; TMT-B η^2^ = 0.01, *p-value* = 1.63e-08; DSP-FW η^2^ = 0.003, *p-value* < 0.001; DSP-BCK η^2^ = 0.006, *p-value* = 1.43e-05; and, DST η^2^ = 0.005, *p-value* < 0.001), confirming their contributions to a cEF latent factor similar to the one proposed by Hatoum^22^. Visit was significantly associated with TMT-A (η^2^ = 0.01, *p* = 3.29e-09), TMT-B (η^2^ = 0.004, *p* = 0.001), and DST (η^2^ = 0.01, *p* = 1.23e-07). Importantly, no interactions between the PGS-cEF and visit were observed with any individual EF tests. A visual depiction of these results is presented in Fig. 3a. A summary of all effect sizes and p-values is presented in eTable 8, and a visual representation of the R^2^ of the models is found in eFigure 2. The random intercepts calculated for each diagnostic group are summarized and presented in the eTable 9.

### No impact of the PGS-PF on phenotypic cEF scores over time

Phenotypic latent cEF scores were only associated with visit (η^2^ =0.03, *p* < 1.32e-15) in the model adjusted for PGS-PF, and not with the PGS-PF or the interaction between visit and PGS-PF. Visit was also associated with TMT-A scores (η^2^ = 0.003, p-value = 0.01). All the individual tests showed no association with the PGS-PF. No association between the interaction of PGS-PF and visit was observed either for the cEF or for any of the individual tests (Fig. 3b). A summary of the effect sizes and p-values is presented in eTable 10, and the trend of the R^2^ values is presented in eFigure 3. The random intercepts calculated for each diagnostic group are summarized and presented in the eTable 11.

## Discussion

In this study we evaluated the influence of polygenic scores (PGS) on practice effects of executive functions testing. We found that PGS for executive functioning (PGS-cEF) differed according to broad diagnostic groups being higher in controls and lower in patients with affective and psychotic disorders, in that order of intensity. In contrast, PGS for general psychopathology (PGS-PF) presented the inverse trend for each of those groups. Moreover, the PGS-cEF were associated with the longitudinal scores of the latent cEF and interacted with time (study visit). This interaction effect was not observed when testing the PGS-PF.

### Effects of repeated cognitive testing on performance

The latent cEF scores showed a positive trend over the four study visits. This finding is consistent with previous research, which has shown that tests of cognitive ability tend to improve over time. A meta-analysis of repeated cognitive test scores collected from 122 studies and 174 samples found that the second test scores improved by 0.3 SD, the third test scores improved by 0.5 SD, and the fourth test scores reached a plateau^9^. In contrast, in our study we observed a constant linear improvement in latent cEF scores during the four study visits for both controls and patients. This could be due to the shorter inter-test period used in our study. The meta-analysis reported a mean inter-test interval of 25.36 weeks (approx. 6 months) between the first and fourth test, while in our study this interval was 18 months. However, we also observed lower improvements in patients with schizophrenia, which is consistent with previous reports^40^. The fact that patients with psychiatric disorders do not benefit from retesting as much as controls could be mediated by a lower genetic predisposition for executive functioning. This would suggest that patients with psychiatric disorders may have a different underlying cognitive profile that affects their ability to improve on cognitive tests over time. Future studies focusing on cognitive performance over time in patients with psychotic disorders could test this hypothesis.

### Scores of the phenotypic latent cEF and PGS are different according to diagnosis

Controls and patients with affective disorders exhibited higher mean phenotypic cEF scores than patients with psychotic disorders, consistent with previous findings^41^. Notably, controls also presented higher PGS for cEF scores, which are indicative of better performance. This raises the question of whether a genetic predisposition for reduced EF could increase the risk for psychiatric disorders, a topic that requires further investigation. In contrast, the PGS-PF scores exhibited an inverse trend compared to the cEF scores, a phenomenon that has been previously reported as inversely associated and transdiagnostic in psychiatric disorders^27^. For example, a study using polygenic scores for ADHD, major depressive disorder, schizophrenia, bipolar disorder, and autism from a sample of children clinically diagnosed with ADHD found a significant association between the scores and one principal component extracted from EF tests of inhibitory control (Stroop test), cognitive flexibility (trail-making test), and digit span (working memory)^42^.

### Polygenic scores for cEF interact with time to influence the longitudinal latent cEF scores

The primary objective of our study was to investigate whether PGS-cEF predict performance improvements after repeated testing. Our results showed that the interaction of PGS-cEF and time (“visit”) was significantly associated with latent cEF scores, providing evidence for the first time regarding the relationship between genetic predisposition and practice effects after repeated EF testing^18^. In contrast, a previous study that followed older adults for 4 years found no changes in the executive function domain, and its association with a PGS for general cognition was not significant^43^. It is important to highlight that EF and general cognition despite being genetically correlated, are two separable constructs both at the phenotypic and genetic level^44^. This underlines the importance of using a broader construct definition when studying EF and preferably calculating the latent cEF. Importantly, no PGS: Visit interaction was found for any of the single EF tests, demonstrating again the unity and diversity structure of EF. Besides sharing variability, the latent cEF shows different psychometric characteristics, as well as genetic correlates^3^. While our results are encouraging regarding short-term repeated EF testing, we cannot conclude that PGS for a cEF predicts improvement effects over longer periods of time^45^.

### Psychopathology genetic liability did not influence longitudinal EF

Our study found that overall genetic liability to psychopathology (PGS-PF), although related to EF scores, did not affect performance improvements. The genetic liability for psychiatric disorders has been previously studied in association with cognitive performance and related brain regions.

Previous studies have shown that genetic liability for psychiatric disorders is associated with cognitive impairment. For example, analysis of polygenic scores for schizophrenia revealed an association with cognitive impairment in patients with schizophrenia, their relatives, and healthy controls^46^. Similarly, analysis of candidate genes from a GWAS of MDD showed genes related with synaptic structure and neurotransmission, that were enriched in the pre-frontal cortex^47^. A longitudinal study found that the specific genetic liability of schizophrenia in healthy older adults was associated with general cognition at baseline, but not with changes in the 10-year follow up measurements^48^. This suggests that genetic liability for schizophrenia may be associated with cognitive function, but not with changes in cognitive function over time. However, different PGS for specific psychiatric disorders were not associated with executive functions^49^. This suggests that the relationship between genetic liability for psychiatric disorders and executive functions may be complex and requires further investigation. Further analysis using the cEF construct is needed for both cross-sectional and longitudinal assessments of cognition in participants with diverse psychopathology genetic predisposition, as patients themselves or their relatives. This will help to better understand the relationship between genetic liability for psychiatric disorders and cognitive function.

### Limitations and future directions

Our study provides insights into the association between genetics and EF performance improvement over an 18-month timeframe. However, there are several limitations to consider. Firstly, a longer follow-up period would be beneficial to observe the influence of genetic predisposition during aging in these patients, which is recommended to future researchers to gain a more comprehensive understanding of this relationship.

The broad age range (18–86 years) complicates interpretation of cognitive change. Executive function shows a canonical developmental trajectory: rapid maturation through mid-adolescence, stabilization in young adulthood, and progressive decline in older age^50^. Although our models control for age, this approach has limitations. In younger participants, cognitive improvements may partially reflect ongoing executive maturation rather than practice effects alone, though effects within 18 months are likely minimal. In older participants, age-related decline may obscure practice benefits. Additionally, cognitive gains could reflect recovery toward baseline in treatment-responsive individuals rather than pure practice effects; however, our interim clinical data showed stable psychiatric symptoms without substantial improvement or decline. Future age-stratified studies would clarify developmental versus pathological contributions to cognitive change.

A key limitation of our study is the modest predictive power of currently available polygenic scores (PGS) for complex cognitive phenotypes such as executive function practice effects. PGS aggregate thousands of common variants with individually small effect sizes, capturing only a fraction of SNP-based heritability and remaining agnostic to the biological function of contributing loci^51^. Further GWAS with large sample sizes of longitudinal cognitive change in psychiatric populations could refined PGS approaches to better elucidate the genetic architecture behind practice gains; moreover, functional validation experiments could bring more light about the contribution of specific genes to longitudinal executive functioning. Consequently, the absence of robust PGS associations at the level of individual EF tests aligns with prior work showing limited explanatory power of PGS for single cognitive tasks, which highlights the advantage of calculating a latent factor.

Repeated testing of executive function is common in clinical and research-oriented psychiatric settings. Our results suggest, although with small size effects, that the potential to gain from repeated testing is related with the genetic liability (polygenic scores) for executive functioning, meaning that some individuals with or without psychiatric diagnoses will obtain higher scores over time independently of additional interventions or disease modification. In this line, calculating PGS for executive functioning might aid to discern if score gains - or the lack of them - are due to inherently genetic predisposition (PGS) or to exogenous factors (interventions/disease progression). Further research is needed to better understand the potential role of applying PGS for executive functioning in clinical or research routines, whether this implementation improves personalized care, and which is the optimal test-retest interval.

Another limitation is that the PGS for the “P factor” refers to broad psychopathology risk, instead of informing as to what the genetic risk for specific psychopathology traits is. Further studies with specific PGS for psychiatric disorders could add more information about the risk of executive dysfunction within these patients.

Additionally, the predicted scores were constructed from the available data in the four visits, which may not be representative of the entire sample. Specifically, the analysis was limited to participants who had at least one test score available from at least one visit. particularly given the relatively low retention rate of 38.8% at the fourth visit.

A study with a larger sample size and lower attrition rates would be optimal to test additional potential confounders and to replicate the results of the current study. This would also allow for a more robust analysis of the relationship between genetics and EF performance improvement. Finally, the sample consisted exclusively of participants of European genetic ancestry, which reduces the generalizability of the results to the majority of the world’s population. This limitation should be considered when interpreting the results, and future studies should aim to recruit a more diverse sample to increase the external validity of the findings.

## Conclusions

Our study provides a first observational exploration on the association of genetic predisposition and EF performance improvements. We found that both patients with psychotic and affective disorders, as well as controls, improved after repeated testing although at different magnitudes, and that PGS-cEF were significantly associated with performance improvement. Including PGS-cEF information and calculating a latent construct could provide additional information regarding the potential for benefiting from practice effects after repeated EF testing, which together with other related factors as disease status and cognitive reserve could bring a broader clinical understanding of patients with psychiatric disorders. Further validation in larger and geographically diverse populations, as well as adjustment by other confounders is needed.

## Supplementary Information

Below is the link to the electronic supplementary material.

Supplementary Material 1

## Data Availability

The data that support the findings of this study are available on request from the corresponding author, UH. The data are not publicly available due to restrictions from the German Data Protection Rules for genetic data, aiming to safeguard the privacy of research participants. The code created for the analysis of these results is available upon request from the corresponding author.

## Abbreviations

AIC: Akaike information criterion
BIC: Bayesian information criterion
cEF: Common executive function factor
CFI: Comparative fit index
DSP-BCK: Digit span backwards
DSP-FW: Digit span forwards
DST: Digit-symbol test
EF: Executive functions
GWAS: Genome-wide association studies
PGS: Polygenic scores
PF: Psychopathology “p” factor
PPD: Patients with psychiatric disorders
SNPs: Single nucleotide polymorphisms
STROBE-ME: Strengthening the Reporting of Observational studies in Epidemiology – Molecular Epidemiology
TMT-A: Trail Making Test A
TMT-B: Trail Making Test B
