# Supplementary material for: Polygenic scores for executive functioning as predictors of performance improvements after repeated testing in major psychiatric disorders
Source: Sci Rep. 2026 Mar 16;16:9199. doi: 10.1038/s41598-026-41345-1 (PMC12996620; doi:10.1038/s41598-026-41345-1)
Supplement: Supplementary file 1 — Supplementary Material 1 [file 41598_2026_41345_MOESM1_ESM.docx]

**This is the Supplementary Material of**

**“****Polygenic scores for executive functioning as predictors of performance improvements after repeated testing in major psychiatric disorders”**

Navarro-Flores, Alba, MD; Heilbronner, Maria, MSc; Rafiee, Hajar, MSc; Wendel, Bernadette, PhD; Papiol, Sergi, PhD; Adorjan, Kristina, MD; Budde, Monika, PhD; Oraki Kohshour, Mojtaba, PhD; Schulte, Eva C., MD, PhD; Reich-Erkelenz, Daniela, MA; Senner, Fanny, MD; Anghelescu, Ion-George, MD; Arolt, Volker, MD; Baune, Bernhard T., MD; Dannlowski, Udo, MD, Dr. Phil; Dietrich, Detlef E., MD; Fallgatter, Andreas J., MD; Figge, Christian, MD; Lang, Fabian U., MD; Juckel, Georg, MD; Konrad, Carsten, MD; Reimer, Jens, MD; Reininghaus, Eva Z., MD; Schmauß, Max, MD; Schmitt, Andrea, MD; Spitzer, Carsten, MD; Wiltfang, Jens,MD; Zimmermann, Jörg, MD; Falkai, Peter, MD; Schulze, Thomas G., MD; Heilbronner, Urs, PhD

**Content**

[**eTable1.** **Checklist** 2](#_Toc220103987)

[**eTable2.** **Sub-diagnostic groups included and their sample sizes** 6](#_Toc220103988)

[**eMethods1.** **Genotyping and Quality Control of the samples** 7](#_Toc220103989)

[**eMethods2.** **Calculation of the latent phenotypic cEF score** 7](#_Toc220103990)

[**eMethods3.** **Polygenic scores** 8](#_Toc220103991)

[**eMethods4.** **Description of the Equations applied** 9](#_Toc220103992)

[**eFigure1.** **Correlation between the individual neuropsychological test scores** 10](#_Toc220103993)

[**eTable3.** **Overview of time effects** 11](#_Toc220103994)

[**eTable4.** **Comparison of invariance models for the construction of the latent cEF phenotypic scores** 11](#_Toc220103995)

[**Supplementary Results. Evaluation of attrition** 12](#_Toc220103996)

[**eTable5.** **Summary of variables at Visit 2** 14](#_Toc220103997)

[**eTable6.** **Summary of variables at Visit 3** 16](#_Toc220103998)

[**eTable7.** **Summary of variables at Visit 4** 18](#_Toc220103999)

[**eTable8.** **Summary of estimates of the models for the PGS-cEF** 20](#_Toc220104000)

[**eTable10.** **Summary of estimates of the models for the PGS-PF** 21](#_Toc220104001)

[**eTable11.** **Random intercepts for diagnostic groups in the PGS-PF models** 21](#_Toc220104002)

[**eFigure2.** **R2 of the different test scores of the PS-cEF model** 22](#_Toc220104003)

[**eFigure3.** **R2 of the different test scores of the PS-PF models** 22](#_Toc220104004)

[**References** 23](#_Toc220104005)

1. **Checklist**

| **Section/Topic** | **Item** | **STROBE guidelines** | **ME extension** | **Pages** |
| --- | --- | --- | --- | --- |
| **Title and abstract** | 1 | (a) Indicate the design of the study with a commonly used term in the title or the abstract | ME-1 State the use of specific biomarker(s) in the title and/or abstract if they contribute substantially to the findings | 1, 4 |
|  |  | (b) Provide in the abstract an informative and balanced summary of what was done and what was found |  | 4 |
| **Introduction** | | | |  |
| Background rationale | 2 | Explain the scientific background and rationale for the investigation being reported | ME-2 Explain in the scientific background of the paper how/why the specific biomarker(s) have been chosen, potentially among many others (e.g. others are studied but reported elsewhere or not studied at all) | 6-7 |
| Objectives | 3 | State specific objectives, including any pre-specified hypotheses | ME-3 *A priori* hypothesis: if one or more biomarkers are used as proxy measures, state the *a priori* hypothesis on the expected values of the biomarker(s) | 7 |
| **Methods** | | | |  |
| Study design | 4 | Present key elements of study design early in the paper | ME-4 Describe the special study designs for molecular epidemiology (in particular, nested case/control and case/cohort) and how they were implemented | 7 |
| *Biological sample collection* |  |  | ME-4·1 Report on the setting of the biological sample collection; amount of sample; nature of collecting procedures; participant conditions; time between sample collection and relevant clinical or physiological endpoints | Supp |
| *Biological sample storage* |  |  | ME-4·2 Describe sample processing (centrifugation, timing, additives, etc.) | Supp |
| *Biological sample processing* |  |  | ME-4·3 Describe sample storage until biomarker analysis (storage, thawing, manipulation, etc.) | Supp |
| *Biomarker biochemical characteristics* |  |  | ME-4·4 Report the half-life of the biomarker and chemical and physical characteristics (e.g. solubility) | Supp |
| Setting | 5 | Describe the setting, locations and relevant dates, including periods of recruitment, exposure, follow-up and data collection |  | 7 |
| Participants | 6 | (a) Cohort study – Give the eligibility criteria and the sources and methods of selection of participants. Describe methods of follow-up | ME-6 Report any habit, clinical condition, physiological factor or working or living condition that might affect the characteristics or concentrations of the biomarker | NA |
|  |  | Case–control study – Give the eligibility criteria and the sources and methods of case ascertainment and control selection. Give the rationale for the choice of cases and controls |  | NA |
|  |  | Cross-sectional study – Give the eligibility criteria and the sources and methods of selection of participants |  | NA |
|  |  | (b) Cohort study – For matched studies, give matching criteria and number of exposed and unexposed |  | 7 |
|  |  | Case–control study – For matched studies, give matching criteria and the number of controls per case |  | NA |
| Variables | 7 | Clearly define all outcomes, exposures, predictors, potential confounders and effect modifiers. Give diagnostic criteria, if applicable |  | 8 |
| Data source/ measurement | 8 | For each variable of interest, give sources of data and details of methods of assessment (measurement). Describe comparability of assessment methods if there is more than one group | ME-8 Laboratory methods: report type of assay used, detection limit, quantity of biological sample used, outliers, timing in the assay procedures (when applicable) and calibration procedures or any standard used | 8,9 |
| Bias | 9 | Describe any efforts to address potential sources of bias |  | 9 |
| Study size | 10 | Explain how the study size was arrived at. |  | 7 |
| Quantitative variables | 11 | Explain how quantitative variables were handled in the analyses. If applicable, describe which groupings were chosen and why |  | 9 |
| Statistical methods | 12 | (a) Describe all statistical methods, including those used to control for confounding | ME-12 Describe how biomarkers were introduced into statistical models | 9 |
|  |  | (b) Describe any methods used to examine subgroups and interactions |  | 9 |
|  |  | (c) Explain how missing data were addressed |  | 9 |
|  |  | (d) Cohort study – If applicable, how loss to follow-up was addressed |  | 9 |
|  |  | Case–control study – If applicable, explain how matching of cases and controls was addressed |  | NA |
|  |  | Cross-sectional study – If applicable, describe analytical methods taking account of sampling strategy |  | NA |
|  |  | (e) Describe any sensitivity analyses |  | 9 |
| *Validity/reliability of measurement and internal/external validation* |  |  | ME-12·1 Report on the validity and reliability of measurement of the biomarker(s) coming from the literature and any internal or external validation used in the study | NA |
| **Results** | | | |  |
| Participants | 13 | (a) Report the numbers of individuals at each stage of the study – e.g. numbers potentially eligible, examined for eligibility, confirmed eligible, included in the study, completing follow-up and analysed | ME-13 Give reason for loss of biological samples at each stage | 9 |
|  |  |  | (b) Give reasons for nonparticipation at each stage | NA |
|  |  |  | (c) Consider use of a flow diagram | NA |
| Descriptive data | 14 | (a) Give characteristics of study participants (e.g. demographic, clinical and social) and information on exposures and potential confounders |  | 9 |
|  |  | (b) Indicate the number of participants with missing data for each variable of interest |  | 9 |
|  |  | (c) Cohort study – Summarize follow-up time (e.g. average and total amount) |  | 9 |
| *Distribution of biomarker measurement* |  |  | ME-14·1 Give the distribution of the biomarker measurement (including mean, median, range and variance) | 9 |
| Outcome data | 15 | Cohort– Numbers of outcome events or summary measures over time |  | 9 |
|  |  | Case–control– Report numbers in each exposure category or summary measures of exposure |  | NA |
|  |  | Cross-sectional– Report numbers of outcome events or summary measures |  | NA |
| Main results | 16 | (a) Give unadjusted estimates and, if applicable, confounder-adjusted estimates and their precision (e.g. 95% confidence interval). Make clear which confounders were adjusted for and why they were included |  | 9-11 |
|  |  | (b) Report category boundaries when continuous variables were categorized |  | NA |
|  |  | (c) If relevant, consider translating estimates of relative risk into absolute risk for a meaningful time period |  | NA |
| Other analyses | 17 | Report other analyses done – e.g. analyses of subgroups and interactions and sensitivity analyses |  | 9-11 |
| **Discussion** | | | |  |
| Key results | 18 | Summarize key results with reference to study objectives |  | 11 |
| Limitations | 19 | Discuss study limitations, considering sources of potential bias/imprecision. Discuss both direction and magnitude of any potential bias. | ME-19 Describe main limitations in laboratory procedures | 14 |
| Interpretation | 20 | Give a cautious overall interpretation of results considering objectives, limitations, multiplicity of analyses, results from similar studies and other relevant evidence | ME-20 Give an interpretation of results in terms of *a priori* biological plausibility | 11-14 |
| Generalizability | 21 | Discuss the generalizability (external validity) of the study results |  | 14 |
| **Other information** | | | |  |
| Funding | 22 | Give the source of funding and the role of the funders for the present study and, if applicable, for the original study on which the present article is based |  | 15 |
| Ethics |  |  | ME-22·1 Describe informed consent and approval from ethical committee(s). Specify whether samples were anonymous, anonymized or identifiable | 8 |

***Legend.*** *Gallo V, Egger M, McCormack V, et al. STrengthening the Reporting of OBservational studies in Epidemiology - Molecular Epidemiology (STROBE-ME): an extension of the STROBE statement [published correction appears in Eur J Clin Invest. 2012 Apr;42(4):462]. Eur J Clin Invest. 2012;42(1):1-16. doi:10.1111/j.1365-2362.2011.02561.x*

1. **Sub-diagnostic groups included and their sample sizes**

| **Diagnosis (SCID)** | **n°** |
| --- | --- |
| **Healthy controls** | **394** |
| **Psychotic Disorders** | **561** |
| Schizophrenia |  |
| 295.10 | 13 |
| 295.20 | 10 |
| 295.30 | 377 |
| 295.60 | 4 |
| 295.90 | 9 |
| F20* | 45 |
| Schizophreniform disorder |  |
| 295.40 | 10 |
| Brief psychotic disorder |  |
| 298.80 | 6 |
| Schizoaffective disorder |  |
| 295.70 | 87 |
| **Affective Disorders** | **603** |
| Recurrent Major Depression |  |
| 296.3 | 94 |
| Bipolar II Disorder |  |
| 296.89 | 112 |
| Bipolar I Disorder |  |
| 296.XX | 397 |

**Legend.** Adult participants (≥18 years) were recruited into clinical and control groups. Clinical

participants were selected based on ICD-10 diagnoses of schizophrenia (F20.X), acute and transient psychotic disorder (F23.X), schizoaffective disorder (F25.X), bipolar disorder (F31.X), manic episode (F30.X), or recurrent depressive disorder (F33.X). DSM-IV diagnoses were confirmed during the baseline study visit using the Structured Clinical Interview for DSM-IV (SCID). Eligible participants met one of the following DSM-IV diagnostic criteria: schizophrenia (295.1/.2/.3/.6/.9), schizophreniform disorder (295.4), brief psychotic disorder (298.8), schizoaffective disorder (295.7), bipolar disorder (296.X), or recurrent major depression (296.3). Participants whose DSM-IV diagnosis derived from the SCID interview deviated from these predefined categories were excluded. * This study participants come from a small subset of the cohort called “MImicSS”. These are PsyCourse participants, but they were recruited using a modified protocol, in which their ICD-10 diagnoses were not reassessed within the DSM-IV framework. All of these clinical participants have ICD-10 Schizophrenia (F20.0).

1. **Genotyping and Quality Control of the samples**

Veinous blood samples were collected during each visit and DNA was extracted. The genotyping was done using the Illumina Infinium Global Screening Array-24 Kit (GSA Array, version 1 and 3; Illumina, San Diego, CA). (1)

Low-quality SNPs and samples were filtered, duplicates and outliers were excluded, as well as sex chromosomes, and SNPs that did not meet specific criteria for Hardy-Weinberg equilibrium and population frequency expectations in European reference populations (SNPs with call rates <98% or a minor allele frequency (MAF) <0.5%). For an in-depth description of the QC see Solomon et al. (1)

After quality control, the total number of individuals was 1,600 and the number of SNPs was 428,907. Imputation was conducted using the Michigan Imputation Server (imputationserver.sph.umich.edu), (2) by comparing allele frequencies in the Haplotype Reference Consortium (HRC) reference panel, in order to identify and remove genetic variants with an outstanding difference in frequency (>20%), or those which did not match the HRC panel regarding position or alleles. Finally, the imputed dataset contained N=1,594 individuals and 7,712,287 SNPs. The Haplotype Reference Consortium (version r1.1 2016) (hg19) was applied as the post-imputation reference genome. (3) For n=6 genotyped individuals, no phenotype data were available, resulting in a final sample size of n=1,594.

1. **Calculation of the latent phenotypic cEF score**

The calculation of the scores of the latent common EF factor (cEF) was analogous to Hatoum et al. (4) We included a classic EF test, the Trail Making Test (Parts A and B), as well as other measures from which EF components could be extracted, following the Unity and Diversity framework of EFs. (5) Those were the Digit-Symbol test, the Digit-Span Forward test and the Digit-Span Backward test (explained below). These tests were assessed paper-based, along with a battery of questionnaires relevant for psychiatric research, at each of the four study visits: visit 1 (T1; baseline), 2 (T2; +6 months), 3 (T3; +12 months), and 4 (T4; +18 months) (see Budde et al,(6)):

- **Trail Making Test A:** Participants are required to connect 25 numbers in ascending order. The time for completion is the score. Errors were immediately corrected by the interviewer. Scores greater than 300 seconds were set to 300. This part of the test is considered to measure visual search and motor speed. (7) Data were reverse log-transformed to fulfill the requirement of normal distribution.
- **Trail Making Test B:** In this test, participants need to connect 25 numbers and letters in alternating ascending (numerical or alphabetical) order. The time for completion is the score. Errors were immediately corrected by the interviewer. Scores greater than 300 seconds were set to 300. The domains measured are suggested to be cognitive flexibility and executive control. (7) Data were reverse log-transformed to fulfill the requirement of normal distribution.
- **Digit Symbol Test:** Participants are required to match symbols to numbers following a legend presented in the upper part of the page. The numbers are presented in rows, with empty rows below them for the writing of the symbols. The time for completion is set to 120 seconds, and the number of correct symbols written is the score. It tests proper motor speed, attention, and visuoperceptual functions, which require high executive control. (8)
- **Digit-Span Forward:** This test (as well as the Backward version) is a component of the Wechsler Adult Intelligence Scale III Test. (9) It consists of the participant’s verbal repetition of digits in the same order as enounced by the examiner. The sequences start at a length of two digits and increase in length after successful completion, with two trials for each digit length. The final score is the sum of all correctly recalled trials. The test mainly assesses attention, immediate verbal recall, and short-term memory capacity, which relate to cognitive control and components of focused attention and resistance to distraction, skills needed for proper executive performance.
- **Digit-Span Backward:** Like the Digit-Span Forward test, digits are presented verbally to a participant, but the participant is required to recite them in the reverse order. The sequences start at a length of two digits and increase in length after successful completion, with two trials for each digit length. The final score is the sum of all correctly recalled trials. This test focuses on verbal working memory, requiring EF for the mental process of reversing the order of the digits.

1. **Polygenic scores**

For the calculation of the polygenic scores for the common executive function factor (PGS-cEF), the summary statistics (training samples) were obtained from the GWAS of Hatoum et al. after personal communication. (4) Briefly, in that study, a sample from the UK Biobank along with the neuropsychological evaluations (latent cEF scores) of a total of 427,037 individuals of European ancestry was included. For the PGS for the p-factor, the summary statistics were obtained from Grotzinger et al. (10) The PGS were derived for PsyCourse participants (target sample). QC included the removal of SNPs that were ambiguous or that had mismatching alleles. Then, the corrected posterior weights were computed using the *phi=auto* setting of the software PRS-CS. (11)The number of individual SNPs that were used to construct the PGS-cEF were 1,070,009; and 871,704 for the PGS-PF. The European UK Biobank was used as the LD reference panel.

1. **Description of the Equations applied**

The equations of the statistical models applied for both models, either PGS-cEF and PGS-PF were based on this equation:

$${EF}_{ij}=\beta_{0}+\sum_{p=1}^{9} \beta_{p}X_{p,ij}+ u_{\frac{center}{patient}}+ v_{diagnosis}+ \epsilon_{ij}$$

Where:

- EFij = latent common executive function (cEF) factor scores or individual EF test score for participant i at visit j
- β_0_ = fixed intercept (population mean EF scores)
- β_p_ = fixed effect coefficient for predictor p (p = 1, 2, . . . , 9)
- Xp,ij = predictor values for participant i at visit j, including:

1. Age: Covariate of interest

2. Sex: Confounder

3. First principal component (PC1): Confounder

4. Second principal component (PC2): Confounder

5. Third principal component (PC3): Confounder

6. Fourth principal component (PC4): Confounder

7. Polygenic score (PGS): Main independent variable, either PGS-cEF or PGS-PF

8. Visit number: Covariate of interest

9. PGS × Visit interaction term

- i = participant index
  - j = visit/assessment timepoint index
  - $u_{\frac{center}{patient}}$ = random intercept for center nested within patient (accounts for clustering of repeated measures within individuals and individuals within recruitment centers); u ∼ N(0, σ^2^ u)
- $v_{diagnosis}$ = random intercept for diagnostic group (accounts for between-diagnosis variation); v ∼ N(0, σ^2^ v)
  - $\epsilon_{ij}$ = residual error term for participant i at visit j; $\epsilon\sim N\left( 0, \sigma_{\epsilon}^{2} \right)$

1.
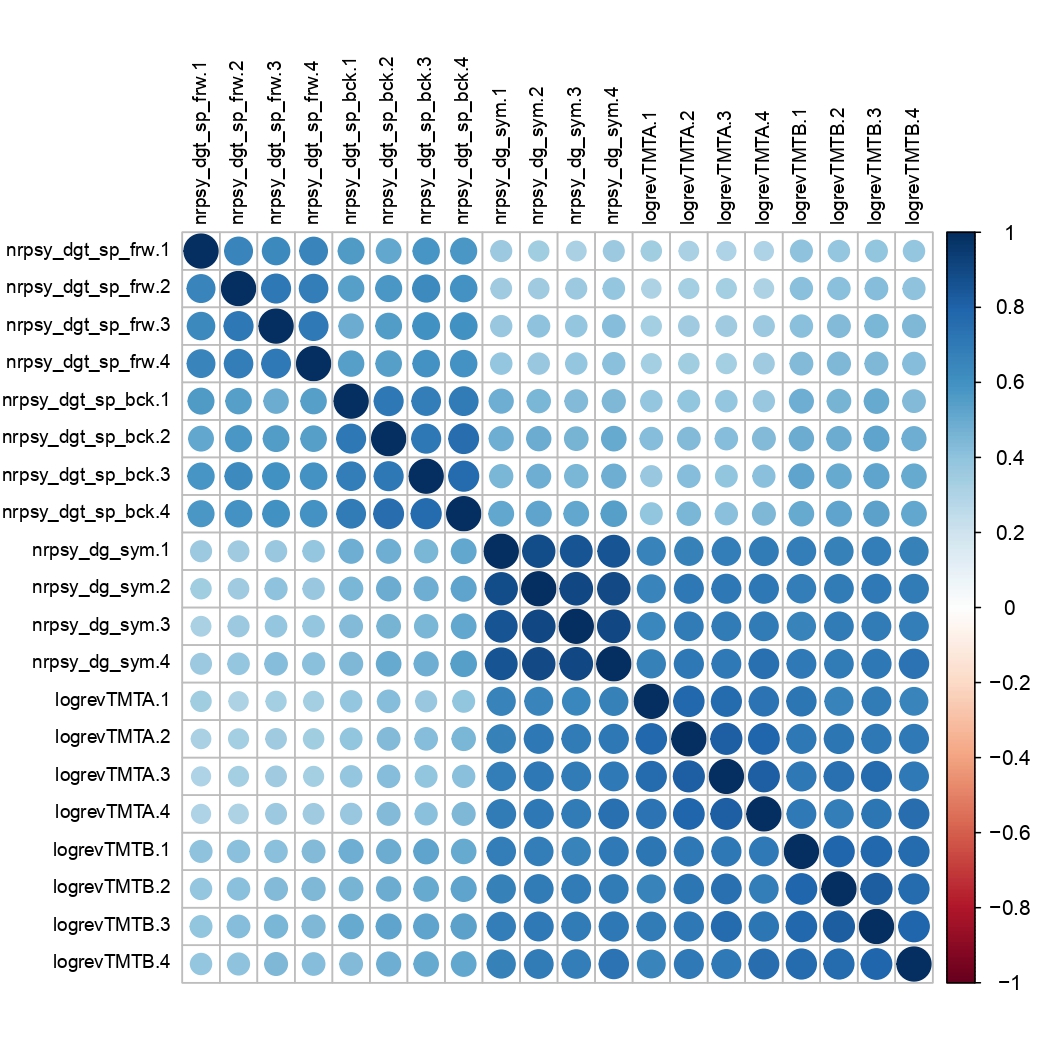
**Correlation between the individual neuropsychological test scores**

**Legend.** Nrpsy_dgt_sp_frw: digit span forward test, Nrpsy_dgt_sp_bck: digit span backwards test, Nrpsy_dg_sym: digit symbol test, logrevTMTA: reverse logarithm of the Trail Making Test-A, logrevTMTB: reverse logarithm of the Trail Making Test-B. Suffix digits form 1 to 4 indicate the number of the study visit.

# **Overview of time effects**

| Variable | Transform | Eta2 | 95% CI | p |
| --- | --- | --- | --- | --- |
| latent cEF | - | 0.88 | 0.87 - 1.00 | <2e-16 |
| TMT-A | Reverse log | 0.13 | 0.11 - 1.00 | <2e-16 |
| TMT-B | Reverse log | 0.09 | 0.07 - 1.00 | <2e-16 |
| DSP-FRW | - | 0.008 | 0.00 - 1.00 | 4.71e-06 |
| DSP-BCK | - | 0.03 | 0.02 - 1.00 | <2e-16 |
| DST | - | 0.15 | 0.13 - 1.00 | <2e-16 |

**Legend.** One-sided confidence intervals, upper side fixed at 1.00

1. **Comparison of invariance models for the construction of the latent cEF phenotypic scores**

|  | DF | CFI | AIC | BIC | Chisq | RMSEA | P value |
| --- | --- | --- | --- | --- | --- | --- | --- |
| Configural | 134 | 0.965 | 38556 | 39070 | 781.78 |  |  |
| Weak | 146 | 0.964 | 38557 | 39007 | 807.24 | 0.026835 | 0.01278 * |
| Strong | 158 | 0.962 | 38581 | 38967 | 855.14 | 0.043816 | 3.262e-06 *** |
| Strict | 173 | 0.961 | 38586 | 38891 | 889.85 | 0.029044 | 0.00270 ** |

**Legend.** DF: degrees of freedom, CFI: comparative fit index, AIC: Akaike information criterion, BIC: Bayesian information criterion, Chisq: Chi squared statistic, RMSEA: Root Mean Square Error of Approximation.

# **Supplementary Results. Evaluation of attrition**

To evaluate potential selection bias of this longitudinal analysis we performed a post-hoc comparison between the baseline scores of those who attend the assessments once vs. more than once. We analyze differences in sociodemographic variables (sex, age, educational attainment), clinical characteristics (General Assessment of Function, medication use, polygenic scores for the common executive function factor [cEF] and for the p-factor) and cognitive measures (latent cEF scores, and individual test scores).

Participants with affective disorders who dropped out were not different than those assess twice or more in any of the aforementioned variables. Patients with psychotic disorders who had two assessments or more were significative older (42 vs 35) and use less tranquilizers. However, their GAF scores were not significantly different, which shows their overall functionality was similar. Only one individual test score (DSB) was statistically significantly higher in those who had more than two test assessments (median 6 vs 5), although that difference is not clinically relevant. The rest of the test cognitive scores did not differ between those who dropped out and the ones assessed repeatedly. We concluded that these two groups did not have cognitive relevant differences. In the case of the control group, the participants who dropped out were older. Clinically not relevant but statistically significant differences were found for the latent cEF scores, and the scores of TMT-A, DSB, and DST, although within expected in the general population. Based on this evidence, the potential bias of the attrition influencing the direction of the results to show participants with “higher cognitive gains” does not hold. Overall, we conclude that the attrition was due to Missing At Random (MAR) and not due to inherent cognitive advantages.

For the analysis of the study, we used Linear Mixed Models which applied Full Information Maximum Likelihood (FIML). LMMs do not assume that those who dropped out are identical to those who came back; rather, they assume the data is MAR. This means the model uses the available data from early waves, where the dropouts were present, to estimate the trajectory they likely would have followed. By incorporating the intercept and early slopes of these individuals, the model partially corrects for the 'super-performer' bias in the remaining sample. This is particularly important for the models including the individual test scores.

**Controls**

| **Characteristics** | **One visit** | **≥ 2 visits** | **p-value***^2^* |
| --- | --- | --- | --- |
|  | N = 109*^1^* | N = 285*^1^* |  |
| **Female** | 60 (55%) | 171 (60%) | 0.4 |
| **Age** | 28 (24, 37) | 33 (24, 52) | 0.013 |
| **Educational Attainment** | 7 (3, 7) | 6 (2, 7) | 0.4 |
| **GAF** | 100 (90, 100) | 88 (84, 91) | <0.001 |
| Missing | 71 | 79 |  |
| **Latent cEF scores** | 1.32 (0.87, 1.86) | 1.23 (0.62, 1.72) | 0.036 |
| **TMT-A** | 23 (18, 28) | 25 (19, 32) | 0.045 |
| **TMT-B** | 51 (40, 65) | 55 (44, 68) | 0.067 |
| **DSB** | 9 (7, 11) | 7 (6, 9) | <0.001 |
| **DSF** | 11 (10, 13) | 10 (9, 12) | 0.012 |
| **DST** | 0.86 (0.39, 1.28) | 0.67 (0.11, 1.23) | 0.025 |
| **PGS-cEF** | 0.20 (0.14, 0.25) | 0.21 (0.16, 0.25) | 0.2 |
| **PGS-PF** | -1.14 (-1.19, -1.09) | -1.14 (-1.20, -1.09) | >0.9 |
| *^1^* n (%); Median (Q1, Q3) | | | |
| *^2^* Pearson’s Chi-squared test; Wilcoxon rank sum test; Fisher’s exact test | | | |

**Affective Disorders**

| Characteristics | One visit | ≥ 2 visits | p-value*^2^* |
| --- | --- | --- | --- |
|  | N = 175*^1^* | N = 416*^1^* |  |
| Female | 94 (54%) | 210 (50%) | 0.5 |
| Age | 45 (34, 55) | 47 (33, 55) | >0.9 |
| Educational Attainment | 5 (2, 7) | 5 (1, 7) | 0.8 |
| GAF | 60 (51, 69) | 60 (51, 70) | 0.8 |
| Antidepressants | 1 (0, 3) | 1 (0, 3) | 0.5 |
| Antipsychotics | 1 (0, 5) | 1 (0, 5) | 0.6 |
| Mood stabilizers | 1 (0, 3) | 1 (0, 3) | 0.8 |
| Tranquilizers | 0 (0, 2) | 0 (0, 2) | 0.2 |
| Other psychiatric medication | 0 (0, 1) | 0 (0, 2) | 0.4 |
| Latent cEF scores | 0.32 (-0.42, 0.92) | 0.37 (-0.22, 0.94) | 0.3 |
| TMT-A | 34 (25, 41) | 31 (24, 41) | 0.3 |
| TMT-B | 73 (52, 103) | 74 (54, 100) | >0.9 |
| DSB | 6 (5, 7) | 6 (5, 7) | 0.063 |
| DSF | 9 (8, 11) | 9 (8, 11) | 0.4 |
| DST | -0.27 (-0.78, 0.30) | -0.27 (-0.78, 0.39) | >0.9 |
| PGS-cEF | 0.20 (0.15, 0.24) | 0.20 (0.16, 0.25) | 0.4 |
| PGS-PF | -1.12 (-1.19, -1.08) | -1.13 (-1.19, -1.08) | 0.7 |
| *^1^* n (%); Median (Q1, Q3) | | | |
| *^2^* Pearson’s Chi-squared test; Wilcoxon rank sum test; Fisher’s exact test | | | |

**Psychotic Disorders**

| **Characteristics** | **One visit** | **≥ 2 visits** | **p-value***^2^* |
| --- | --- | --- | --- |
|  | N = 184*^1^* | N = 349*^1^* |  |
| **Female** | 62 (34%) | 139 (40%) | 0.2 |
| **Age** | 35 (27, 47) | 42 (32, 50) | <0.001 |
| **Educational Attainment** | 4 (1, 7) | 4 (1, 7) | 0.09 |
| **GAF** | 51 (43, 60) | 55 (45, 62) | 0.065 |
| **Antidepressants** | 0 (0, 2) | 0 (0, 2) | 0.2 |
| **Antipsychotics** | 2 (0, 5) | 2 (0, 4) | 0.2 |
| **Mood stabilizers** | 0 (0, 1) | 0 (0, 2) | 0.2 |
| **Tranquilizers** | 0 (0, 2) | 0 (0, 2) | <0.001 |
| **0** | 119 (65%) | 279 (80%) |  |
| **1** | 59 (32%) | 63 (18%) |  |
| **2** | 6 (3.3%) | 7 (2.0%) |  |
| **Other psychiatric medication** | 0 (0%) | 4 (1.1%) | 0.3 |
| **Latent cEF scores** | -0.07 (-0.56, 0.41) | -0.02 (-0.63, 0.55) | 0.8 |
| **TMT-A** | 35 (27, 45) | 36 (29, 48) | 0.092 |
| **TMT-B** | 85 (65, 114) | 84 (64, 110) | 0.8 |
| **DSB** | 5 (4, 6) | 6 (4, 7) | 0.025 |
| **DSF** | 9 (7, 10) | 9 (8, 11) | 0.12 |
| **DST** | -0.64 (-1.20, -0.22) | -0.64 (-1.11, -0.08) | 0.3 |
| **PGS-cEF** | 0.18 (0.14, 0.22) | 0.19 (0.15, 0.24) | 0.064 |
| **PGS-PF** | -1.10 (-1.16, -1.03) | -1.11 (-1.17, -1.06) | 0.10 |
| *^1^* n (%); Median (Q1, Q3) | | | |
| *^2^* Pearson’s Chi-squared test; Wilcoxon rank sum test; Fisher’s exact test | | | |

**Legend of the tables.** GAF: General Assessment of Function, cEF: Common Executive Function Factor, TMT-A: Trail making test A, TMT-B: Trail making test B, DSB: Digit Span Backward, DSF: Digit Span Forward, DST: Digit Symbol Test.

1. **Summary of variables at Visit 2**

|  | **Total (N=954)** | **Controls (N=251)** | **Affective (N=369)** | **Psychotic (N=334)** |
| --- | --- | --- | --- | --- |
| **Females** | 470 (49.3%) | 152 (60.6%) | 183 (49.6%) | 135 (40.4%) |
| **Age** |  |  |  |  |
| Mean (SD) | 43.3 (14.0) | 39.4 (16.1) | 46.1 (13.5) | 43.2 (11.9) |
| **Educational attainment** |  |  |  |  |
| Mean (SD) | 4.97 (1.58) | 5.67 (1.37) | 5.07 (1.54) | 4.34 (1.53) |
| Median [Min, Max] | 5.00 [1.00, 7.00] | 6.00 [2.00, 7.00] | 5.00 [1.00, 7.00] | 4.00 [1.00, 7.00] |
| Missing | 21 (2.2%) | 4 (1.6%) | 11 (3.0%) | 6 (1.8%) |
| **GAF** |  |  |  |  |
| Mean (SD) | 67.8 (16.2) | 87.2 (7.21) | 65.5 (12.9) | 59.3 (14.2) |
| Median [Min, Max] | 68.0 [28.0, 100] | 89.0 [61.0, 100] | 65.0 [30.0, 98.0] | 59.0 [28.0, 98.0] |
| Missing | 74 (7.8%) | 63 (25.1%) | 6 (1.6%) | 5 (1.5%) |
| **No. of Antidepressants** |  |  |  |  |
| Mean (SD) | 0.368 (0.608) | 0.00398 (0.0631) | 0.634 (0.722) | 0.347 (0.547) |
| Median [Min, Max] | 0 [0, 4.00] | 0 [0, 1.00] | 1.00 [0, 4.00] | 0 [0, 3.00] |
| **No. of Antipsychotics** |  |  |  |  |
| Mean (SD) | 0.888 (0.972) | 0 (0) | 0.824 (0.840) | 1.63 (0.884) |
| Median [Min, Max] | 1.00 [0, 5.00] | 0 [0, 0] | 1.00 [0, 5.00] | 2.00 [0, 5.00] |
| **No. of Mood stabilizers** |  |  |  |  |
| Mean (SD) | 0.317 (0.531) | 0.0159 (0.125) | 0.664 (0.595) | 0.159 (0.419) |
| Median [Min, Max] | 0 [0, 3.00] | 0 [0, 1.00] | 1.00 [0, 3.00] | 0 [0, 2.00] |
| **No. of other Psych. medication** |  |  |  |  |
| Mean (SD) | 0.0126 (0.121) | 0 (0) | 0.0298 (0.186) | 0.00299 (0.0547) |
| Median [Min, Max] | 0 [0, 2.00] | 0 [0, 0] | 0 [0, 2.00] | 0 [0, 1.00] |
| **Tranquilizers** |  |  |  |  |
| Mean (SD) | 0.0901 (0.304) | 0.0159 (0.125) | 0.106 (0.317) | 0.129 (0.369) |
| Median [Min, Max] | 0 [0, 2.00] | 0 [0, 1.00] | 0 [0, 2.00] | 0 [0, 2.00] |
| **cEF score** |  |  |  |  |
| Mean (SD) | 0.763 (1.09) | 1.58 (0.914) | 0.653 (0.992) | 0.270 (0.976) |
| Median [Min, Max] | 0.826 [-2.84, 3.69] | 1.65 [-1.15, 3.69] | 0.746 [-2.84, 2.94] | 0.336 [-2.39, 2.74] |
| **TMT-A score** |  |  |  |  |
| Mean (SD) | 31.9 (15.2) | 24.3 (9.51) | 33.1 (16.1) | 36.4 (15.5) |
| Median [Min, Max] | 28.0 [10.0, 142] | 22.0 [10.0, 71.0] | 29.0 [12.0, 142] | 33.0 [14.0, 103] |
| Missing | 3 (0.3%) | 0 (0%) | 2 (0.5%) | 1 (0.3%) |
| **TMT-B score** |  |  |  |  |
| Mean (SD) | 74.0 (38.2) | 55.2 (23.9) | 77.9 (39.5) | 84.9 (40.6) |
| Median [Min, Max] | 64.0 [22.0, 300] | 51.0 [22.0, 225] | 69.0 [24.0, 300] | 75.0 [22.0, 263] |
| Missing | 49 (5.1%) | 4 (1.6%) | 14 (3.8%) | 31 (9.3%) |
| **Digit Span Backwards** |  |  |  |  |
| Mean (SD) | 6.53 (2.26) | 7.75 (2.30) | 6.37 (2.02) | 5.76 (2.09) |
| Median [Min, Max] | 6.00 [0, 14.0] | 7.00 [2.00, 14.0] | 6.00 [2.00, 14.0] | 6.00 [0, 13.0] |
| Missing | 16 (1.7%) | 1 (0.4%) | 6 (1.6%) | 9 (2.7%) |
| **Digit Span Forwards** |  |  |  |  |
| Mean (SD) | 9.69 (2.20) | 10.5 (2.01) | 9.51 (2.19) | 9.25 (2.18) |
| Median [Min, Max] | 10.0 [4.00, 16.0] | 11.0 [4.00, 15.0] | 10.0 [4.00, 15.0] | 9.00 [5.00, 16.0] |
| Missing | 13 (1.4%) | 0 (0%) | 4 (1.1%) | 9 (2.7%) |
| **Digit-Symbol test** |  |  |  |  |
| Mean (SD) | -0.0118 (0.983) | 0.771 (0.838) | -0.0687 (0.860) | -0.553 (0.808) |
| Median [Min, Max] | -0.0324 [-2.66, 3.11] | 0.812 [-2.66, 3.11] | -0.0793 [-2.10, 2.41] | -0.595 [-2.33, 1.98] |
| Missing | 24 (2.5%) | 1 (0.4%) | 13 (3.5%) | 10 (3.0%) |

**Legend.** Table containing participants who presented empirical data on at least one neuropsychological test. cEF: latent common Executive Function factor; GAF: Global assessment of Functioning; PS-cEF: Polygenic Scores for the cEF; PS-P factor: Polygenic scores for the P factor. TMT-A: Trail Making Test, part A; TMT-B: Trail Making Test, part B. Educational attainment was calculated as a composite score of basic and higher education years.

1. **Summary of variables at Visit 3**

|  | **Total (N=839)** | **Controls (N=246)** | **Affective (N=310)** | **Psychotic (N=283)** |
| --- | --- | --- | --- | --- |
| **Sex** |  |  |  |  |
| Female | 422 (50.3%) | 148 (60.2%) | 159 (51.3%) | 115 (40.6%) |
| Males | 417 (49.7%) | 98 (39.8%) | 151 (48.7%) | 168 (59.4%) |
| **Age** |  |  |  |  |
| Mean (SD) | 43.8 (14.2) | 38.9 (15.9) | 47.4 (13.6) | 44.1 (11.7) |
| **Educational attainmentᵃ** |  |  |  |  |
| Mean (SD) | 5.01 (1.61) | 5.70 (1.39) | 5.12 (1.55) | 4.26 (1.55) |
| Median [Min, Max] | 5.00 [1.00, 7.00] | 6.00 [2.00, 7.00] | 5.00 [1.00, 7.00] | 4.00 [1.00, 7.00] |
| Missing | 19 (2.3%) | 2 (0.8%) | 10 (3.2%) | 7 (2.5%) |
| **GAF** |  |  |  |  |
| Mean (SD) | 67.9 (16.8) | 87.1 (8.70) | 64.7 (13.3) | 58.7 (14.1) |
| Median [Min, Max] | 68.0 [28.0, 100] | 90.0 [50.0, 100] | 65.0 [32.0, 98.0] | 58.0 [28.0, 98.0] |
| Missing | 69 (8.2%) | 62 (25.2%) | 6 (1.9%) | 1 (0.4%) |
| **No. of Antidepressants** |  |  |  |  |
| Mean (SD) | 0.347 (0.572) | 0.00407 (0.0638) | 0.613 (0.686) | 0.353 (0.521) |
| Median [Min, Max] | 0 [0, 3.00] | 0 [0, 1.00] | 1.00 [0, 3.00] | 0 [0, 2.00] |
| **No. of Antipsychotics** |  |  |  |  |
| Mean (SD) | 0.870 (0.982) | 0 (0) | 0.839 (0.851) | 1.66 (0.886) |
| Median [Min, Max] | 1.00 [0, 5.00] | 0 [0, 0] | 1.00 [0, 5.00] | 2.00 [0, 5.00] |
| **No. of Mood stabilizers** |  |  |  |  |
| Mean (SD) | 0.305 (0.517) | 0.0163 (0.127) | 0.684 (0.583) | 0.141 (0.388) |
| Median [Min, Max] | 0 [0, 3.00] | 0 [0, 1.00] | 1.00 [0, 3.00] | 0 [0, 2.00] |
| **No. of other Psych. medication** |  |  |  |  |
| Mean (SD) | 0.0131 (0.124) | 0 (0) | 0.0323 (0.194) | 0.00353 (0.0594) |
| Median [Min, Max] | 0 [0, 2.00] | 0 [0, 0] | 0 [0, 2.00] | 0 [0, 1.00] |
| **No. of Tranquilizers** |  |  |  |  |
| Mean (SD) | 0.108 (0.340) | 0.00813 (0.0900) | 0.139 (0.373) | 0.163 (0.415) |
| Median [Min, Max] | 0 [0, 2.00] | 0 [0, 1.00] | 0 [0, 2.00] | 0 [0, 2.00] |
| **TMT-A score** |  |  |  |  |
| Mean (SD) | 30.9 (15.6) | 23.0 (9.21) | 32.8 (17.3) | 35.7 (15.6) |
| Median [Min, Max] | 27.0 [9.00, 179] | 20.0 [9.00, 63.0] | 28.0 [12.0, 179] | 32.0 [13.0, 106] |
| Missing | 4 (0.5%) | 1 (0.4%) | 1 (0.3%) | 2 (0.7%) |
| **TMT-B score** |  |  |  |  |
| Mean (SD) | 73.9 (42.4) | 53.3 (24.6) | 77.7 (42.7) | 88.2 (47.5) |
| Median [Min, Max] | 64.0 [23.0, 300] | 46.0 [23.0, 175] | 68.0 [31.0, 300] | 76.0 [30.0, 300] |
| Missing | 21 (2.5%) | 2 (0.8%) | 9 (2.9%) | 10 (3.5%) |
| **Digit Span Backwards** |  |  |  |  |
| Mean (SD) | 6.57 (2.40) | 7.80 (2.41) | 6.27 (2.20) | 5.81 (2.18) |
| Median [Min, Max] | 6.00 [1.00, 14.0] | 8.00 [2.00, 14.0] | 6.00 [2.00, 12.0] | 5.00 [1.00, 12.0] |
| Missing | 16 (1.9%) | 1 (0.4%) | 7 (2.3%) | 8 (2.8%) |
| **Digit Span Forwards** |  |  |  |  |
| Mean (SD) | 9.74 (2.23) | 10.8 (2.20) | 9.42 (2.13) | 9.18 (2.03) |
| Median [Min, Max] | 10.0 [3.00, 16.0] | 11.0 [6.00, 16.0] | 9.00 [3.00, 15.0] | 9.00 [4.00, 14.0] |
| Missing | 14 (1.7%) | 0 (0%) | 6 (1.9%) | 8 (2.8%) |
| **Digit-Symbol test** |  |  |  |  |
| Mean (SD) | 0.124 (1.03) | 0.943 (0.824) | -0.00938 (0.890) | -0.482 (0.831) |
| Median [Min, Max] | 0.155 [-2.42, 3.11] | 0.906 [-1.30, 3.11] | 0.0145 [-2.42, 2.36] | -0.501 [-2.28, 2.12] |
| Missing | 25 (3.0%) | 0 (0%) | 8 (2.6%) | 17 (6.0%) |

**Legend.** Table containing participants who presented empirical data on at least one neuropsychological test. cEF: latent common Executive Function factor; GAF: Global assessment of Functioning; PS-cEF: Polygenic Scores for the cEF; PS-P factor: Polygenic scores for the P factor. TMT-A: Trail Making Test, part A; TMT-B: Trail Making Test, part B. Educational attainment was calculated as a composite score of basic and higher education years.

1. **Summary of variables at Visit 4**

|  | **Total (N=742)** | **Controls (N=219)** | **Affective (N=249)** | **Psychotic (N=274)** |
| --- | --- | --- | --- | --- |
| **Females** | 352 (47.4%) | 130 (59.4%) | 107 (43.0%) | 115 (42.0%) |
| **Age** |  |  |  |  |
| Mean (SD) | 44.4 (14.0) | 39.7 (15.9) | 48.5 (13.4) | 44.3 (11.7) |
| **Educational attainment** |  |  |  |  |
| Mean (SD) | 4.98 (1.62) | 5.68 (1.39) | 5.14 (1.56) | 4.29 (1.56) |
| Median [Min, Max] | 5.00 [1.00, 7.00] | 6.00 [2.00, 7.00] | 5.00 [1.00, 7.00] | 4.00 [1.00, 7.00] |
| Missing | 12 (1.6%) | 2 (0.9%) | 6 (2.4%) | 4 (1.5%) |
| **GAF** |  |  |  |  |
| Mean (SD) | 67.4 (17.1) | 86.2 (9.07) | 65.8 (13.1) | 57.7 (15.0) |
| Median [Min, Max] | 68.0 [25.0, 99.0] | 89.0 [51.0, 99.0] | 65.0 [35.0, 99.0] | 56.0 [25.0, 95.0] |
| Missing | 60 (8.1%) | 57 (26.0%) | 1 (0.4%) | 2 (0.7%) |
| **No. of Antidepressants** |  |  |  |  |
| Mean (SD) | 0.352 (0.596) | 0.00913 (0.0953) | 0.643 (0.738) | 0.361 (0.539) |
| Median [Min, Max] | 0 [0, 3.00] | 0 [0, 1.00] | 1.00 [0, 3.00] | 0 [0, 2.00] |
| **No. of Antipsychotics** |  |  |  |  |
| Mean (SD) | 0.911 (0.990) | 0 (0) | 0.871 (0.852) | 1.68 (0.865) |
| Median [Min, Max] | 1.00 [0, 5.00] | 0 [0, 0] | 1.00 [0, 5.00] | 2.00 [0, 5.00] |
| **No. of Mood stabilizers** |  |  |  |  |
| Mean (SD) | 0.306 (0.524) | 0.0137 (0.117) | 0.735 (0.584) | 0.150 (0.405) |
| Median [Min, Max] | 0 [0, 3.00] | 0 [0, 1.00] | 1.00 [0, 3.00] | 0 [0, 2.00] |
| **No. of other Psych. medication** |  |  |  |  |
| Mean (SD) | 0.0135 (0.127) | 0 (0) | 0.0402 (0.216) | 0 (0) |
| Median [Min, Max] | 0 [0, 2.00] | 0 [0, 0] | 0 [0, 2.00] | 0 [0, 0] |
| **No. of Tranquilizers** |  |  |  |  |
| Mean (SD) | 0.121 (0.354) | 0.00913 (0.0953) | 0.165 (0.403) | 0.172 (0.415) |
| Median [Min, Max] | 0 [0, 2.00] | 0 [0, 1.00] | 0 [0, 2.00] | 0 [0, 2.00] |
| **TMT-A score** |  |  |  |  |
| Mean (SD) | 29.8 (14.0) | 22.3 (8.28) | 31.4 (15.3) | 34.3 (14.0) |
| Median [Min, Max] | 26.0 [10.0, 117] | 20.0 [10.0, 54.0] | 27.0 [11.0, 117] | 30.0 [13.0, 91.0] |
| Missing | 6 (0.8%) | 0 (0%) | 3 (1.2%) | 3 (1.1%) |
| **TMT-B score** |  |  |  |  |
| Mean (SD) | 72.2 (41.5) | 52.8 (25.4) | 77.8 (43.7) | 83.5 (44.6) |
| Median [Min, Max] | 62.0 [20.0, 300] | 47.0 [20.0, 209] | 69.0 [27.0, 300] | 73.0 [24.0, 300] |
| Missing | 36 (4.9%) | 5 (2.3%) | 9 (3.6%) | 22 (8.0%) |
| **Digit Span Backwards** |  |  |  |  |
| Mean (SD) | 6.80 (2.51) | 8.26 (2.55) | 6.44 (2.19) | 5.91 (2.20) |
| Median [Min, Max] | 6.00 [0, 14.0] | 8.00 [3.00, 14.0] | 6.00 [2.00, 13.0] | 6.00 [0, 12.0] |
| Missing | 16 (2.2%) | 0 (0%) | 3 (1.2%) | 13 (4.7%) |
| **Digit Span Forwards** |  |  |  |  |
| Mean (SD) | 9.66 (2.19) | 10.4 (2.14) | 9.41 (2.17) | 9.25 (2.10) |
| Median [Min, Max] | 10.0 [4.00, 15.0] | 10.0 [4.00, 15.0] | 9.00 [5.00, 15.0] | 9.00 [4.00, 15.0] |
| Missing | 15 (2.0%) | 0 (0%) | 2 (0.8%) | 13 (4.7%) |
| **Digit-Symbol test** |  |  |  |  |
| Mean (SD) | 0.146 (1.06) | 1.02 (0.885) | -0.00997 (0.894) | -0.435 (0.853) |
| Median [Min, Max] | 0.108 [-2.52, 3.11] | 1.05 [-1.44, 3.11] | 0.0145 [-2.47, 2.03] | -0.501 [-2.52, 2.17] |
| Missing | 19 (2.6%) | 2 (0.9%) | 6 (2.4%) | 11 (4.0%) |

**Legend.** Table containing participants who presented empirical data on at least one neuropsychological test. cEF: latent common Executive Function factor; GAF: Global assessment of Functioning; PS-cEF: Polygenic Scores for the cEF; PS-P factor: Polygenic scores for the P factor. TMT-A: Trail Making Test, part A; TMT-B: Trail Making Test, part B. Educational attainment was calculated as a composite score of basic and higher education years.

1. **Summary of estimates of the models for the PGS-cEF**

| **Variable** | **Eta 2** | **p-value** | **p-adjusted** |
| --- | --- | --- | --- |
| **Latent cEF** |  |  |  |
| PRS cEF | 0.03 | 2.25 e-11*** | 1.35e-10*** |
| Visit | 0.39 | < 2.2 e-16*** | <1.32e-15*** |
| PRS cEF:Visit | 0.03 | < 2.2 e-16*** | <1.32e-15*** |
| *R^2^ model: 0.98, fixed: 0.40, random: 0.58* | | | |
| **TMT-A** |  |  |  |
| PRS cEF | 0.008 | 3.79e-07*** | 7.57e-07*** |
| Visit | 0.01 | 1.09e-09*** | 3.29e-09*** |
| PRS cEF:Visit | 5.38e-05 | 0.69 | 0.69 |
| *R^2^ model: 0.79, fixed: 0.27, random: 0.52* | | | |
| **TMT-B** |  |  |  |
| PRS cEF | 0.01 | 5.43e-09*** | 1.63e-08*** |
| Visit | 0.004 | 0.0008*** | 0.001** |
| PRS cEF:Visit | 0.001 | 0.09 | 0.25 |
| *R^2^ model: 0.80, fixed: 0.26, random: 0.54* | | | |
| **DGT-FW** |  |  |  |
| PRS cEF | 0.003 | 0.0007*** | 7.01e-04*** |
| Visit | 0.0003 | 0.34 | 0.34 |
| PRS cEF:Visit | 9.43e-05 | 0.61 | 0.69 |
| *R^2^ model: 0.68, fixed: 0.06, random: 0.62* | | | |
| **DGT-BCK** |  |  |  |
| PRS cEF | 0.006 | 9.56e-06*** | 1.43e-05*** |
| Visit | 0.0007 | 0.15 | 0.18 |
| PRS cEF:Visit | 0.0007 | 0.16 | 0.25 |
| *R^2^ model: 0.73, fixed: 0.08, random: 0.65* | | | |
| **DST** |  |  |  |
| PRS cEF | 0.005 | 0.0002*** | 0.0002*** |
| Visit | 0.01 | 6.15e-08*** | 1.23e-07*** |
| PRS cEF:Visit | 0.0009 | 0.13 | 0.25 |
| *R^2^ model: 0.88, fixed: 0.27, random: 0.61* | | | |

Legend. Adjusted by PC, age, age2, sex. Corrected p values using the FDR method according to variable.

1. **Random intercepts for diagnostic groups in the PGS-cEF models**

| Test | Control | Affective | Psychotic |
| --- | --- | --- | --- |
| cEF | 0.604 | -0.133 | -0.471 |
| TMT-A | 0.174 | -0.037 | -0.138 |
| TMT-B | 0.213 | -0.055 | -0.158 |
| DS-FW | 0.665 | -0.122 | -0.543 |
| DS-BK | 1.116 | -0.366 | -0.751 |
| DST | 0.592 | -0.114 | -0.478 |

1. **Summary of estimates of the models for the PGS-PF**

| **Variable** | **Eta 2** | **p-value** | **p-adjusted** |
| --- | --- | --- | --- |
| **Latent cEF** |  |  |  |
| PRS PF | 0.0004 | 0.38 | 0.76 |
| Visit | 0.03 | < 2.2e-16*** | <1.32e-15*** |
| PRS cEF:Visit | 0.002 | 0.02* | 0.09 |
| *R^2^ model = 0.98, fixed = 0.37, random = 0.61* | | | |
| **TMT-A** |  |  |  |
| PRS PF | 0.002 | 0.03* | 0.09 |
| Visit | 0.003 | 0.004** | 0.01* |
| PRS PF:Visit | 0.0006 | 0.18 | 0.36 |
| *R^2^ model: 0.79, fixed:0.25, random: 0.54* | | | |
| **TMT-B** |  |  |  |
| PRS PF | 0.002 | 0.02 | 0.09 |
| Visit | 0.001 | 0.048 | 0.07 |
| PRS PF:Visit | 0.0002 | 0.44 | 0.44 |
| *R^2^ model: 0.80, fixed: 0.23, random: 0.57* | | | |
| **DGT-FW** |  |  |  |
| PRS PF | 2.56e-05 | 0.78 | 0.78 |
| Visit | 7.16e-04 | 0.16 | 0.19 |
| PRS PF:Visit | 4.06e-04 | 0.29 | 0.43 |
| *R^2^ model:0.68, fixed: 0.04, random: 0.64* | | | |
| **DGT-BCK** |  |  |  |
| PRS PF | 1.22e-03 | 0.77 | 0.78 |
| Visit | 1.87e-03 | 0.03* | 0.06 |
| PRS PF:Visit | 9.05e-04 | 0.15 | 0.36 |
| *R^2^ model:0.73, fixed: 0.05, random: 0.68* | | | |
| **DST** |  |  |  |
| PRS PF | 0.0001 | 0.55 | 0.78 |
| Visit | 0.0002 | 0.51 | 0.51 |
| PRS PF:Visit | 0.0004 | 0.31 | 0.44 |
| *R^2^ model: 0.88, fixed:0.26, random:0.62* | | | |

Legend. Adjusted by PC, age, age^2^, sex. Corrected p values using the FDR method according to variable.

1. **Random intercepts for diagnostic groups in the PGS-PF models**

| Test | Control | Affective | Psychotic |
| --- | --- | --- | --- |
| cEF | 0.625 | -0.138 | -0.487 |
| TMT-A | 0.179 | -0.038 | -0.141 |
| TMT-B | 0.219 | -0.057 | -0.162 |
| DS-FW | 0.716 | -0.125 | -0.591 |
| DS-BK | 1.177 | -0.372 | -0.805 |
| DST | 0.599 | -0.115 | -0.485 |

1. **R2 of the different test scores of the PS-cEF model**


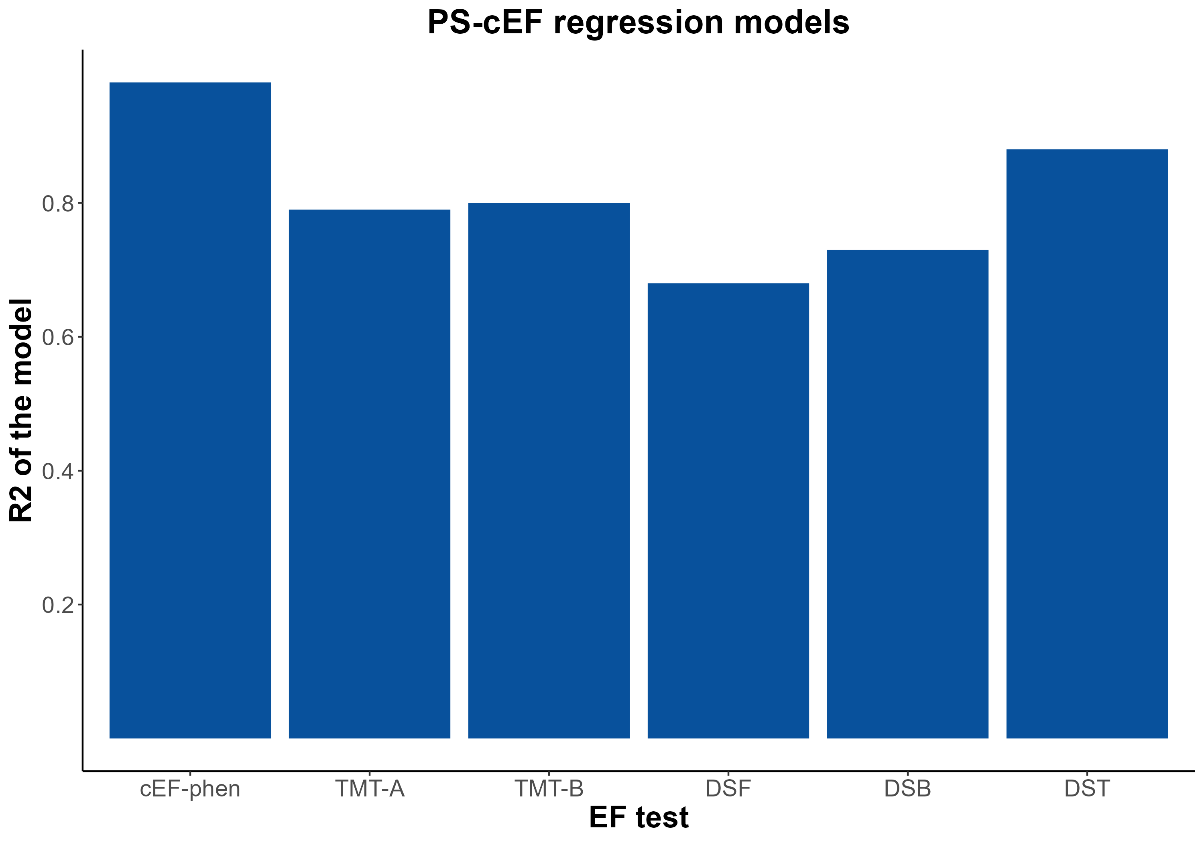


1. **R2 of the different test scores of the PS-PF models**


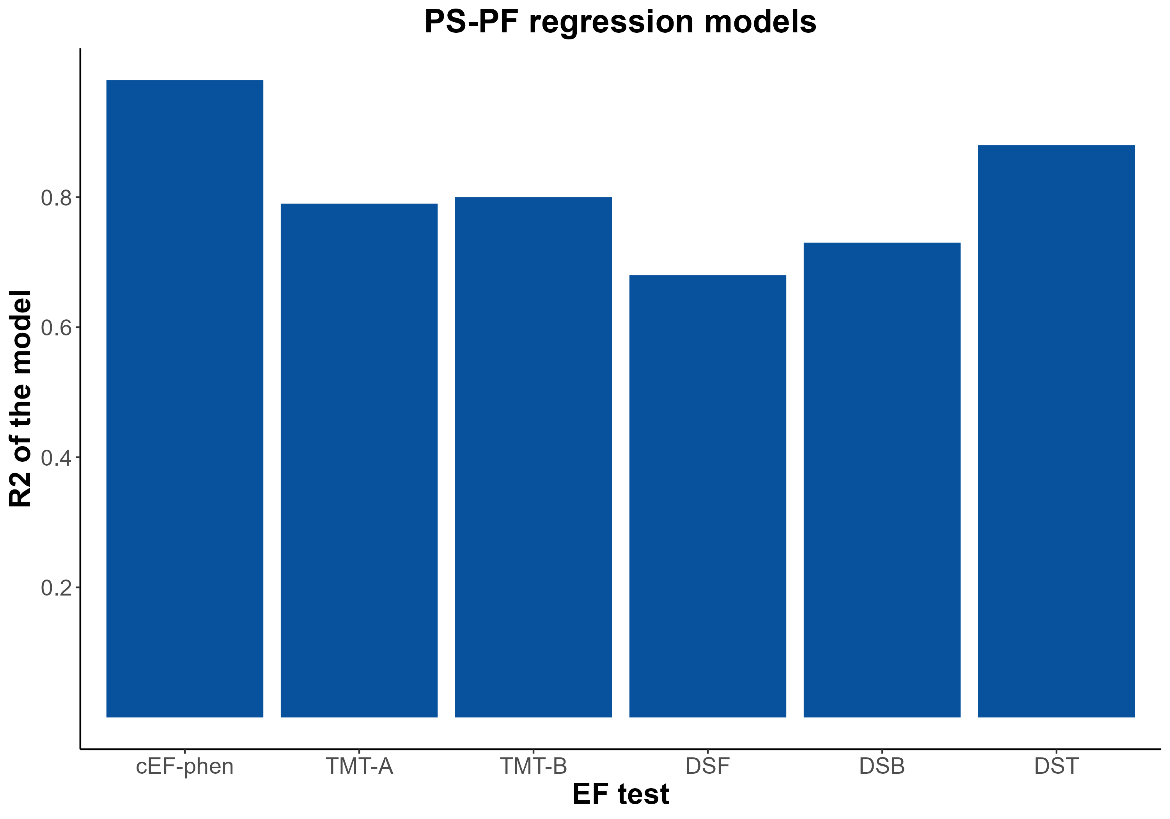


# **References**

1. Solomon P, Kaurani L, Budde M, Guiné JB, Krüger DM, Riquin K, et al. (2025): Integrative analysis of miRNA expression profiles reveals distinct and common molecular mechanisms underlying broad diagnostic groups of severe mental disorders. *Molecular psychiatry*. 30:4364-4383.

2. Das S, Forer L, Schönherr S, Sidore C, Locke AE, Kwong A, et al. (2016): Next-generation genotype imputation service and methods. *Nature genetics*. 48:1284-1287.

3. McCarthy S, Das S, Kretzschmar W, Delaneau O, Wood AR, Teumer A, et al. (2016): A reference panel of 64,976 haplotypes for genotype imputation. *Nature genetics*. 48:1279-1283.

4. Hatoum AS, Morrison CL, Mitchell EC, Lam M, Benca-Bachman CE, Reineberg AE, et al. (2023): Genome-wide Association Study Shows That Executive Functioning Is Influenced by GABAergic Processes and Is a Neurocognitive Genetic Correlate of Psychiatric Disorders. *Biological psychiatry*. 93:59-70.

5. Friedman NP, Miyake A (2017): Unity and diversity of executive functions: Individual differences as a window on cognitive structure. *Cortex; a journal devoted to the study of the nervous system and behavior*. 86:186-204.

6. Budde M, Anderson-Schmidt H, Gade K, Reich-Erkelenz D, Adorjan K, Kalman JL, et al. (2019): A longitudinal approach to biological psychiatric research: The PsyCourse study. *American journal of medical genetics Part B, Neuropsychiatric genetics : the official publication of the International Society of Psychiatric Genetics*. 180:89-102.

7. Bowie CR, Harvey PD (2006): Administration and interpretation of the Trail Making Test. *Nature protocols*. 1:2277-2281.

8. Jaeger J (2018): Digit Symbol Substitution Test: The Case for Sensitivity Over Specificity in Neuropsychological Testing. *Journal of clinical psychopharmacology*. 38:513-519.

9. Wechsler DJ (1997): Wechsler adult intelligence scale-III.

10. Grotzinger AD, Rhemtulla M, de Vlaming R, Ritchie SJ, Mallard TT, Hill WD, et al. (2019): Genomic structural equation modelling provides insights into the multivariate genetic architecture of complex traits. *Nature human behaviour*. 3:513-525.

11. Ge T, Chen CY, Ni Y, Feng YA, Smoller JW (2019): Polygenic prediction via Bayesian regression and continuous shrinkage priors. *Nature communications*. 10:1776.
